# Network meta-analysis to compare the efficacies of three surgical techniques in rheumatic mitral valve disease

**DOI:** 10.1038/s44325-026-00106-9

**Published:** 2026-02-20

**Authors:** Chuang Liu, Song-hao Jia, Mao-zhou Wang, Ming-xuan Zhang, Xiao-long Wang, Hong-jia Zhang, Wen-jian Jiang

**Affiliations:** 1 Department of Cardiovascular Surgery, Beijing Anzhen Hospital, Capital Medical University, Beijing, China; 2 Beijing Laboratory of Cardiovascular Precision Medicine, Beijing Municipal Education Commission, Beijing, China; 3 Key Lab of Medical Engineering for Cardiovascular Disease, Ministry of Education, Beijing, China; 4 Laboratory for Clinical Medicine, Capital Medical University, Beijing, China; 5 Institute of Reproductive and Child Health, National Health Commission Key Laboratory of Reproductive Health, School of Public Health, Peking University Health Science Center, Beijing, China; 6 Department of Epidemiology and Biostatistics, School of Public Health, Peking University Health Science Center, Beijing, China

**Keywords:** Cardiology, Diseases, Medical research

## Abstract

This systematic review compared the efficacies of percutaneous mitral balloon commissurotomy (PMBC), mitral valvuloplasty (MVP), and mitral valve replacement (MVR) in patients with rheumatic mitral valve disease. Data from 15,271 patients across 23 cohort studies and randomized controlled trials were analyzed. Based on the results of the network meta-analysis, MVP demonstrated a lower early mortality rate [odds ratio (OR), 0.71; 95% confidence interval (CI): 0.54–0.92], follow-up mortality rate (OR: 0.84; 95% CI: 0.72-0.99), and complication rate (OR: 0.75; 95% CI: 0.64-0.88) compared to MVR. The follow-up reoperation rate in the MVP group was significantly lower than that in the PMBC group (OR: 0.49; 95% CI: 0.30–0.80). The optimal surgical strategy should be tailored to achieve better prognoses.

## Introduction

Globally, rheumatic heart disease (RHD), a common cardiovascular disease, affects approximately 41 million patients^1^. RHD most commonly affects the mitral valve, causing mitral stenosis^2–5^. The Cape Town Declaration states that progress on the preventive strategies for RHD has been slow with surgery being the only effective treatment for symptomatic RHD^6^. Therefore, it is necessary to develop an optimal surgical strategy for rheumatic mitral stenosis.

The treatment guidelines have been suggested based on percutaneous mitral balloon commissurotomy (PMBC) to delay surgical intervention. Surgical intervention is preferred only when PMBC is contraindicated^7,8^. PMBC surgery is associated with decreased trauma but high reoperation rates^9^. Mitral valve replacement (MVR) is an effective surgical treatment method. However, biological valve replacement has the disadvantage of decreased valve durability, while mechanical valve replacement is associated with anticoagulant-related complications^10^. Several studies have demonstrated that the clinical outcomes of rheumatic mitral valvuloplasty (MVP) technology are superior to those of MVR^11–13^. However, if PMBC is conducted as the initial procedure, the torn commissural often fails to be located at the physiological commissural position. Consequently, this situation markedly increases the difficulty level of the subsequent MVP^14^. Therefore, MVP technology challenges the previous PMBC priority surgical strategy. A comprehensive analysis of previous findings will enable the comparison of the prognosis of these three treatment strategies and the development of an optimal rheumatic mitral valve treatment strategy.

Previous meta-analyses have compared the treatment outcomes of PMBC/MVP or MVP/MVR pairs but not those of the three treatment modalities^15–17^. This study aimed to employ a systematic review approach to analyze cohort studies or randomized controlled trials (RCTs) concerning PMBC/MVP/MVR for the treatment of rheumatic mitral valve disease and conduct a network meta-analysis. The findings of this study will provide evidence for the development of optimal treatment strategies for rheumatic mitral valve disease.

## Results

### Patient Characteristics

**Fig. 1:**
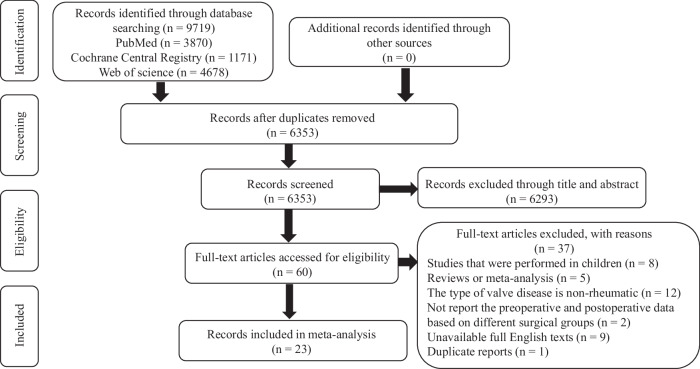
PRISMA flowchart for PMBC, MVP, and MVR. Literature search for network meta-analysis of PMBC, MVP, and MVR. PMBC percutaneous mitral balloon commissurotomy, MVP mitral valvuloplasty, MVRmitral valve replacement, PRISMA Preferred Reporting Items for Systematic Reviews and Meta-Analysis.

An electronic database search revealed 9719 studies (Fig. 1). The data of duplicate and irrelevant studies and studies lacking accessibility of data were censored. Finally, 23 studies involving 15271 patients were included for further analysis. Of these 15271 patients, 894 received PMBC, 2972 received MVP, and 11405 received MVR (Table 1). The data on the main types of mitral valve lesions and the proportion of mechanical valve replacement were extracted. The study characteristics are summarized in Table S1.

**Table 1:** Baseline patient characteristics for the network meta-analysis among PMBC, MVP and MVR

	PMBC	MVP	MVR	*P*-value		
	*n* = 894	*n* = 2972	*n* = 11,405	PMBC vs. MVP	PMBC vs. MVR	MVP vs. MVR
Age, years	42.97 ± 12.84	45.27± 16.18	53.76 ± 14.56	0.80	<0.001	<0.001
Female, %	82.16	68.98	56.32	0.80	<0.001	<0.001
Wilkins score	7.89 ± 1.68	6.50 ± 1.16	9.52 ± 2.20	0.49	<0.001	NR*
MVA, cm^2^	0.95 ± 0.23	1.69 ± 1.17	1.22 ± 0.88	0.48	0.10	<0.001
NYHA III–IV, %	55.47	47.61	52.25	0.32	0.72	<0.001
Atrial fibrillation, %	39.94	53.70	67.88	0.048	<0.001	<0.001

*PMBC* percutaneous mitral balloon commissurotomy, *MVP* mitral valvuloplasty, *MVR* mitral valve replacement, *MVA* mitral valve orifice area, *NYHA* New York Heart Association, *NR* not reported. * The calculation of the *P*-value was based on pairwise comparisons of baseline difference between the two groups. The calculation of the Wilkins score was not feasible, as the literature comparing MVP and MVR failed to report the Wilkins scores for each group.

The study quality assessment is summarized in Tables S2 and S3. The funnel plot did not reveal significant asymmetry to suggest publication bias with early mortality, follow-up mortality, follow-up reoperation and complications (Fig. S1-S4).

**Fig. 2:**
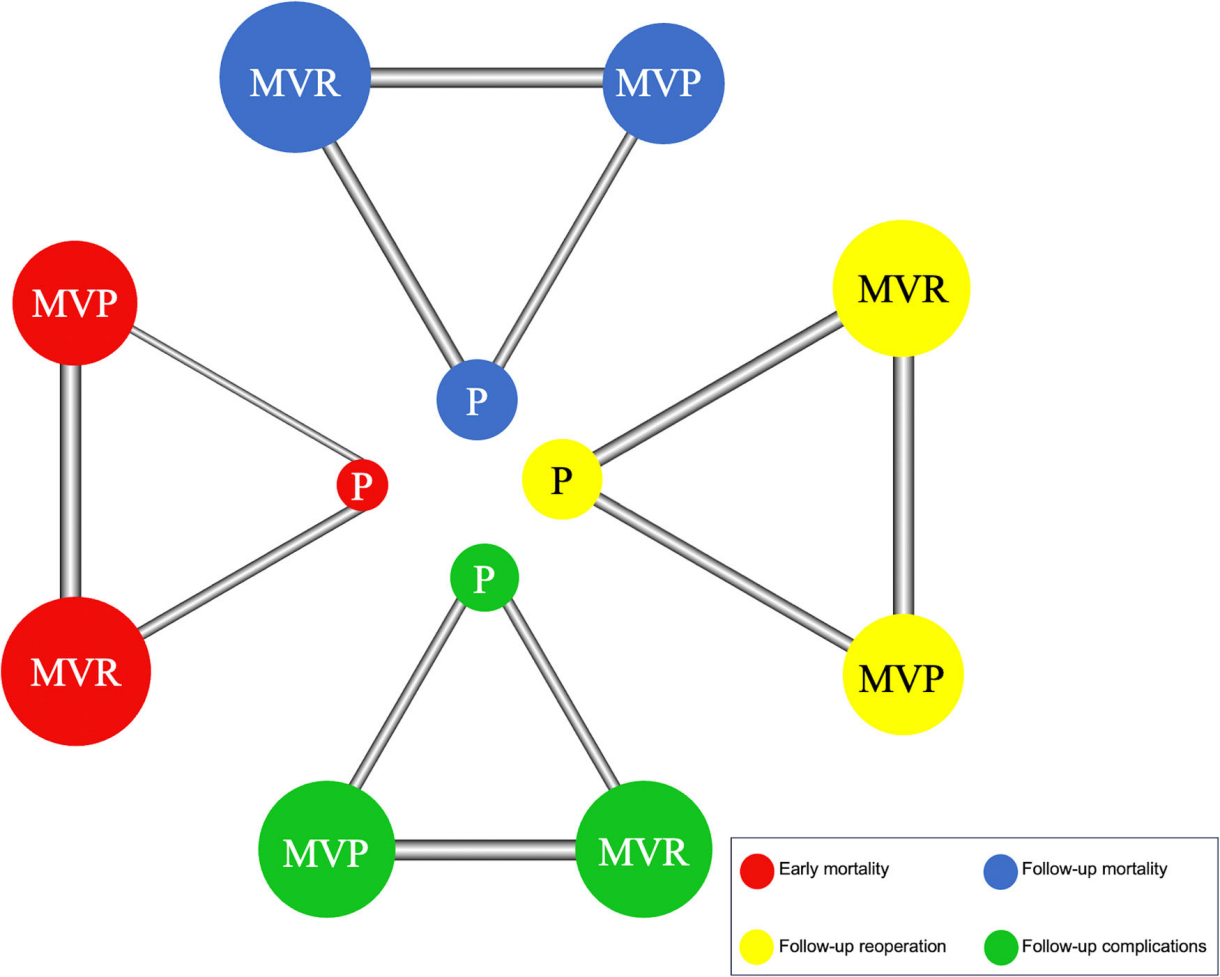
Network diagram for PMBC, MVP, and MVR. Network meta-analysis comparisons for: (A) early mortality, (B) follow-up mortality, (C) follow-up reoperation, (D) follow-up complications. The node size is proportional to the number of researches engaged, and the thickness of the continuous line connecting nodes is proportional to the number of directly comparing researches between the two treatments. P percutaneous mitral balloon commissurotomy, MVP mitral valvuloplasty, MVR mitral valve replacement.

**Fig. 3:**
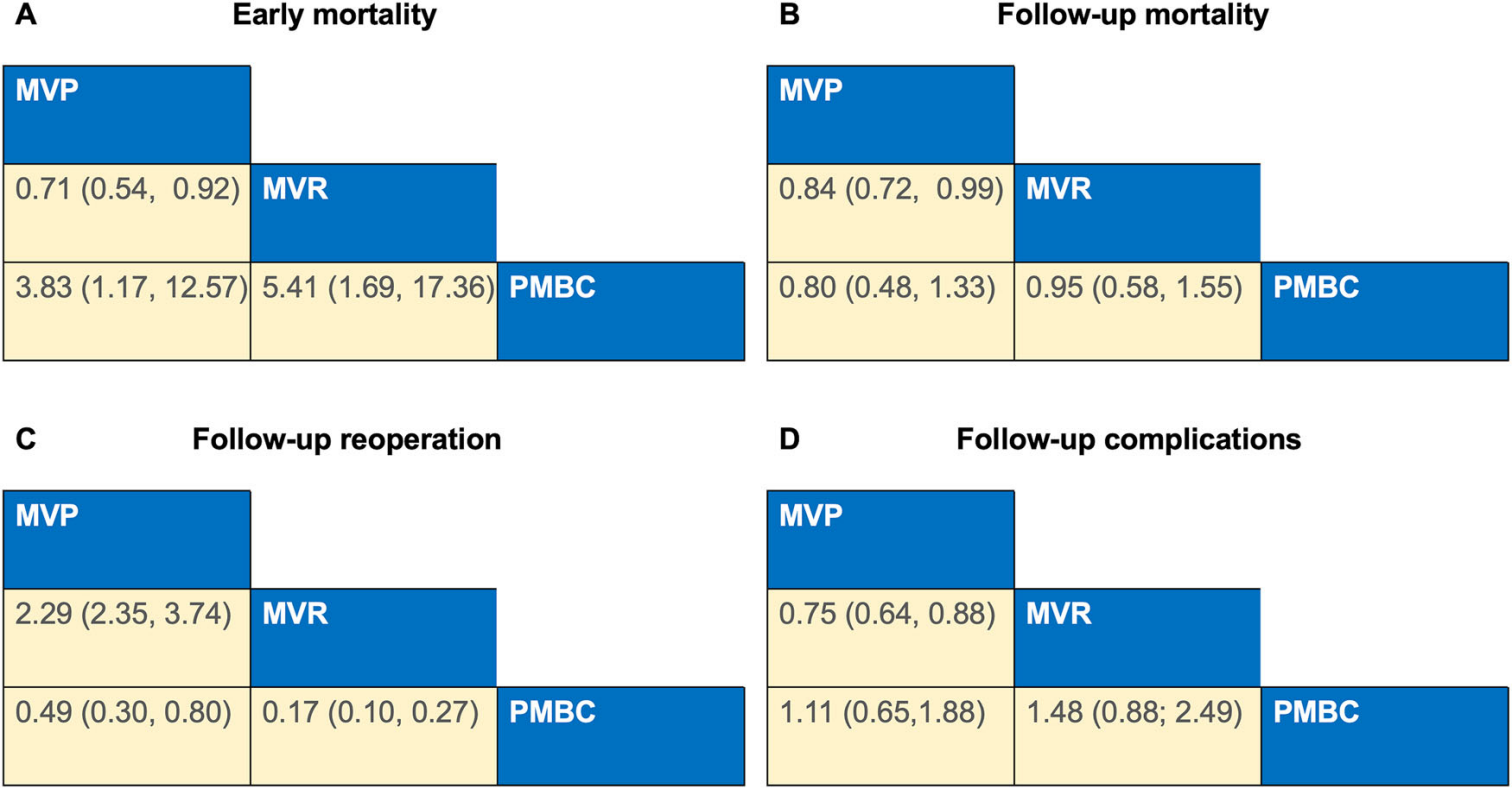
League tables for PMBC, MVP, and MVR. Outcomes shown for (**A**) early mortality, (**B**) follow-up mortality, (**C**) follow-up reoperation, (**D**) follow-up complications following PMBC, MVP, and MVR (odds ratio and 95% confidence interval). Odds ratio < 1 means the treatment in top left is better. PMBC percutaneous mitral balloon commissurotomy, MVP mitral valvuloplasty, MVR mitral valve replacement.

**Fig. 4:**
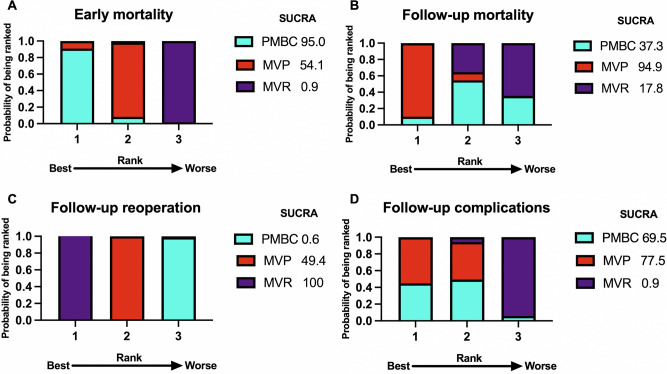
Rankograms for PMBC, MVP and MVR. Outcomes shown for (**A**) early mortality, (**B**) follow-up mortality, (**C**) follow-up reoperation, (**D**) follow-up complications following PMBC, MVP, and MVR. SUCRA, surface under the cumulative ranking curve. PMBC percutaneous mitral balloon commissurotomy, MVP mitral valvuloplasty, MVR mitral valve replacement.

The age (*P* = 0.80), gender (*P* = 0.80), Wilkins score (*P* = 0.49), mitral valve orifice area (MVA) (*P* = 0.48), and New York Heart Association (NYHA) grade (*P* = 0.32) were similar between the MVP and PMBC groups. However, the proportion of patients with atrial fibrillation (AF) (*P* = 0.048) in the MVP group was higher than that in the PMBC group. The baseline characteristics differed significantly between the MVP group and the MVR group. The MVA (*P* = 0.10) and NYHA grade (*P* = 0.72) were similar between the MVR and PMBC groups. Compared with those in the PMBC groups, the age (*P* < 0.001), Wilkins score (*P* < 0.001), proportion of male patients (*P* < 0.001), and proportion of patients with AF (*P* < 0.001) were higher in the MVR group (Table 1). The complete evidence networks, the league tables, and the SUCRA values for all outcomes are shown in Figs. 2, 3A-D, and 4A-D, respectively. The plots of SUCRA values for all outcomes are shown in Fig. S5-S8.

### Early Mortality

Network meta-analysis revealed that the early mortality rate in the MVR group was significantly higher than that in the PMBC group [odds ratio (OR): 5.41; 95% confidence interval (CI): 1.69–17.36]. Meanwhile, the early mortality rate in the MVP group was significantly higher than that in the PMBC group (OR: 3.83; 95% CI: 1.17–12.57). Compared with that in the MVR group, the early mortality rate in the MVP group was significantly lower (OR: 0.71; 95% CI: 0.54–0.92). The league table is shown in Fig. 3A. Analysis of surface under the cumulative ranking curve (SUCRA) values revealed that patients who underwent PMBC had the highest probability of having the lowest early mortality rate (95.0%) (Fig. 4A and Fig. S5). The funnel plot revealed no publication bias in the included studies (Fig. S1).

Next, a pairwise meta-analysis was performed. The early mortality rate in the MVP group was significantly lower than that in the MVR group (OR: 0.63; 95% CI: 0.41–0.96; *I*^*2*^ = 41%, *P*_*heterogeneity*_ = 0.06) (Fig. S9). Meanwhile, the early mortality rate in the PMBC group was significantly lower than that in the MVR group (OR: 0.18, 95% CI: 0.05–0.58; *I*^*2*^ = 0%, *P*_*heterogeneity*_ = 0.91) (Fig. S10). However, the PMBC (1/370, 0.3%) and MVP groups (1/320, 0.3%) exhibited relatively low proportions of early mortality and comprised relatively small sample sizes. Thus, performing a meaningful pairwise meta-analysis between the PMBC and MVP groups was impossible (Fig. S11).

These results indicate that the early mortality rate was as follows: MVR group > MVP group > PMBC group. However, there is a lack of relevant results from direct comparisons regarding the early mortality rates between PMBC and MVP. Therefore, the conclusion that PMBC has a lower early mortality rate than MVP should be interpreted with caution.

### Follow-up Mortality

Network meta-analysis revealed that the follow-up mortality rate in the MVP group was significantly lower than that in the MVR group (OR: 0.84; 95% CI: 0.72–0.99). Meanwhile, the follow-up mortality rate was not significantly different between the MVP and PMBC groups (OR: 0.80; 95% CI: 0.48–1.33), as well as between the MVR and PMBC groups (OR: 0.95; 95% CI: 0.58–1.55). The league table is shown in Fig. 3B. Analysis of SUCRA values revealed that patients who underwent MVP had the highest probability of having the lowest rate of follow-up mortality (94.9%) (Fig. 4B and Fig. S6). The funnel plot did not reveal marked publication bias in the included studies (Fig. S2).

Next, a pairwise meta-analysis was performed. The follow-up mortality rate in the MVP group was significantly lower than that in the MVR group (OR: 0.56; 95% CI: 0.38–0.84; *I*^*2*^ = 73%, *P*_*heterogeneity*_ < 0.01) (Fig. S12). Besides, the follow-up mortality rate was not significantly different between the PMBC and MVP groups (OR: 2.20; 95% CI: 0.72–6.78, *I*^*2*^ = 0%, *P*_*heterogeneity*_ = 0.89) (Fig. S13), as well as between the PMBC and MVR groups (OR: 0.97; 95% CI: 0.58–1.63, *I*^*2*^ = 36%, *P*_*heterogeneity*_ = 0.17) (Fig. S14). Heterogeneity was high between the MVP and MVR groups. In eight studies, the follow-up mortality rate in the MVR group was higher than that in the MVP group. Meanwhile, the follow-up mortality rate in the MVP group was higher than that in the MVR group in only three studies from Taiwan and South Korea, which can be attributed to the heterogeneity of the surgical techniques of MVP. These results indicate that the follow-up mortality rate in the MVR group was higher than that in the MVP group.

### Follow-up Reoperation

Network meta-analysis revealed that the risk of follow-up reoperation in the MVP group was significantly higher than that in the MVR group (OR: 2.29; 95% CI: 2.35–3.74) but lower than that in the PMBC group (OR: 0.49; 95% CI: 0.30–0.80). Meanwhile, the risk of follow-up reoperation in the MVR group was significantly lower than that in the PMBC group (OR: 0.17; 95% CI: 0.10–0.27). The league table is shown in Fig. 3C. Analysis of SUCRA values revealed that patients who underwent MVR had the highest probability of having the lowest rate of follow-up reoperation (100%) (Fig. 4C). The funnel plot did not reveal marked publication bias in the included studies (Fig. S3).

Next, a pairwise meta-analysis was performed. The risk of follow-up reoperation in the MVP group was significantly higher than that in the MVR group (OR: 2.99; 95% CI: 2.02–4.43; *I*^*2*^ = 57%, *P*_*heterogeneity*_ < 0.01) (Fig. S15). Meanwhile, the risk of follow-up reoperation in the PMBC group was significantly higher than that in the MVR group (OR: 6.88; 95% CI: 3.28–14.41; *I*^*2*^ = 32%, *P*_*heterogeneity*_ = 0.19) (Fig. S16). Additionally, the risk of follow-up reoperation in the PMBC group was higher than that in the MVP group (OR: 1.70; 95% CI: 0.85 to 3.43, *I*^*2*^ = 22%, *P*_*heterogeneity*_ = 0.27) (Fig. S17). Heterogeneity was high between the MVP and MVR groups. The risk of follow-up reoperation in the MVP group was higher than that in the MVR group in 11 studies. Meanwhile, the risk of follow-up reoperation in the MVR group was higher than that in the MVP group in only one study from South Korea. These results indicate that the follow-up reoperation risk in different groups was as follows: MVR group < MVP group < PMBC group.

### Follow-up Complications

Network meta-analysis revealed that the risk of follow-up complications in the MVP group was significantly lower than that in the MVR group (OR: 0.75; 95% CI: 0.64–0.88). Meanwhile, the risk of follow-up complications was not significantly different between the MVP and PMBC groups (OR: 1.11; 95% CI: 0.65–1.88), as well as between the MVR and PMBC groups (OR: 1.48; 95% CI: 0.88–2.49). The league table is shown in Fig. 3D. Analysis of SUCRA values revealed that patients who underwent MVP had the highest probability of having the lowest rate of follow-up complications (77.5%) (Fig. 4D). The funnel plot did not reveal marked publication bias in the included studies.

Next, a pairwise meta-analysis was performed. The risk of follow-up complications in the MVP group was significantly lower than that in the MVR group (OR: 0.58; 95% CI: 0.40–0.84; *I*^*2*^ = 72%, *P*_*heterogeneity*_ < 0.01) (Fig. S18). Additionally, the risk of follow-up complications was not significantly different between the PMBC and MVP groups (OR: 1.05; 95% CI: 0.44–2.50, *I*^*2*^ = 0%, *P*_*heterogeneity*_ = 0.96) (Fig. S19), as well as between the PMBC and MVR groups (OR: 0.63; 95% CI: 0.34–1.18, *I*^*2*^ = 0%, *P*_*heterogeneity*_ = 0.45) (Fig. S20). Heterogeneity was high between the MVP and MVR groups. The risk of follow-up complications in the MVP group was lower than that in the MVR group in seven studies. Meanwhile, the risk of follow-up complications in the MVR group was lower than that in the MVP group in only one study from Taiwan. These results indicate that the risk of follow-up complications in the MVP group is lower than that in the MVR group.

### Subgroup Analysis

A subgroup analysis was performed to evaluate the differential early mortality rates and follow-up mortality and reoperation rates in the simple commissurotomy and composite MVP groups relative to the MVR group. The two different MVP groups were not compared with PMBC groups because only one study from China performed a comparative analysis between composite MVP and PMBC groups. The data on the surgical technique of mitral valve repair were extracted (Tables S4 and S5). The surgical techniques of composite MVP were ring annuloplasty, leaflet thinning, commissurotomy, and release of subvalvular apparatus.

This study performed a pairwise meta-analysis. The early mortality rate in the composite MVP group was significantly lower than that in the MVR group (OR: 0.52; 95% CI: 0.30–0.93; *I*^*2*^ = 38%, *P*_*heterogeneity*_ = 0.13) (Fig. S21). However, the early mortality rate was not significantly different between the simple commissurotomy and MVR groups (OR: 0.80;95% CI: 0.29–2.21, *I*^*2*^ = 0%, *P*_*heterogeneity*_ = 0.77) (Fig. S22). The follow-up mortality rate in the composite MVP group was significantly lower than that in the MVR group (OR: 0.51; 95% CI: 0.32–0.80; *I*^*2*^ = 57%, *P*_*heterogeneity*_ = 0.03) (Fig. S23). Meanwhile, the follow-up mortality rate in the simple commissurotomy group was non-significantly lower than that in the MVR group (OR: 0.40;95% CI: 0.16–1.05, *I*^*2*^ = 0%, *P*_*heterogeneity*_ = 0.88) (Fig. S24). The follow-up reoperation rate in the composite MVP group was significantly higher than that in the MVR group (OR: 2.60; 95% CI: 1.58–4.27; *I*^*2*^ = 58%, *P*_*heterogeneity*_ = 0.02) (Fig. S25). Meanwhile, the follow-up reoperation rate in the simple commissurotomy group was significantly higher than that in the MVR group (OR: 4.48; 95% CI: 1.98–10.12; *I*^*2*^ = 0%, *P*_*heterogeneity*_ = 0.48) (Fig. S26). The follow-up complication rate in the composite MVP group was significantly lower than that in the MVR group (OR: 0.51; 95% CI: 0.37–0.71; *I*^*2*^ = 27%, *P*_*heterogeneity*_ = 0.23) (Fig. S27). However, only one study from Italy compared simple commissurotomy with the MVR group and reported that the follow-up complication rate in the simple commissurotomy group was non-significantly lower than that in the MVR group (OR: 0.17; 95% CI: 0.02–1.41) (Fig. S28). Heterogeneity was low.

## Discussion

This is the first network meta-analysis to compare the clinical efficacies of PMBC, MVP, and MVR in rheumatic mitral valve disease. The analysis was performed with 23 cohort studies and RCTs involving 15271 patients. This study aimed to provide new evidence for the development of optimal surgical strategies for rheumatic mitral valve disease. The main findings of this study were as follows: 1) The early mortality rate in the MVR group was higher than that in the MVP and PMBC groups. 2) During the follow-up period, the MVP group exhibited lower mortality and complication rates than the MVR group and a lower reoperation rate than the PMBC group. 3) Surgical techniques for MVP significantly varied. Composite MVP with techniques, such as annuloplasty, leaflet thinning, commissure incision, and subvalvular device release was associated with significantly better outcomes than MVP with only commissurotomy.

RHD is the leading cause of cardiovascular disease and valve disease among young adults in low-income and middle-income countries where more than 80% of the global population resides^1,6^. The mortality rate of RHD is high and is correlated with the severity of valve disease. Valve surgery can significantly mitigate RHD-related mortality^18^. Studies originating from numerous countries have clearly indicated that mitral valve surgery is the predominant surgical approach for patients with rheumatic valve disease.^2–5^. The guidelines in both Europe and the United States advocate for a treatment strategy involving PMBC to delay surgical intervention. Surgery is preferred only when PMBC is contraindicated^7,8^. Advances in MVP technology have challenged the previous PMBC priority surgical strategy. MVP has the great benefit of keeping the patient’s original valve intact. Additionally, valve durability in MVP is higher than that in PMBC^19^. Some experts believe that MVP is the optimal method for treating rheumatic mitral valve disease^20–22^. However, high-quality evidence is currently unavailable to support changing the priority treatment strategy for PMBC. To address this gap, this study conducted a network meta-analysis.

Liao et al.^15^, Jiang et al.^23^, Fu et al.^24^, and Yasmin et al.^16^ performed meta-analyses to compare the outcomes of rheumatic MVP and MVR and reported consistent findings. Rheumatic MVP was beneficial for short-term and mid-term survival. However, the reoperation rate in the MVP group was higher than that in the MVR group, which was consistent with the findings of this study. Singh et al. performed a meta-analysis to compare the efficacies of PMBC and surgical commissurotomy^17^. They reported that PMBC was preferred as it was associated with a decreased peri-procedural morbidity. However, the scope of MVP included in our study is not limited to surgical commissurotomy alone.

Pairwise analysis in this study revealed heterogeneity in the outcomes of MVP. Additionally, this study detailed the surgical methods used in each article in the supplementary table. Some studies used simple incisions at the commissure, while other studies used methods, such as annuloplasty, commissure incision, and leaflet thinning. Subgroup analysis was performed based on the MVP procedures. The procedures were divided into simple commissurotomy and composite MVP. The early mortality rate in the composite MVP group was 48% lower than that in the MVR group (Fig. S21). Furthermore, the follow-up mortality and incidence of complications in the composite MVP group were 49% and 49% lower than those in the MVR group, respectively (Fig. S23 and Fig. S27). However, the mortality rate was not significantly different between the simple commissurotomy and MVR group, and the reoperation rate in the simple commissurotomy group was 4.48 times higher than that in the MVR group. Therefore, comprehensive subgroup analysis revealed that composite MVP was highly effective. The surgical technique used for rheumatic MVP determines the clinical outcomes. A simple commissurotomy may not be an optimal choice for rheumatic MVP.

MVP had the highest probability of having the lowest mortality and complication rates during follow-up (SUCRA values of 94.9% and 77.5%, respectively). Meanwhile, MVR was associated with the lowest reoperation rate (SUCRA value of 100%). PMBC was associated with the lowest early mortality rate (SUCRA value of 95.0%). Our findings indicated that the follow-up reoperation rate of patients undergoing MVP was lower than that of patients undergoing PMBC. Therefore, we recommend that MVP should be prioritized and that MVR should be the least preferred choice among the three treatment options. De Bonis et al. suggested that MVP should be preferred for patients with favorable echocardiographic features^25^. However, currently, MVP and MVR selection mainly relies on the surgeons’ subjective experience during the operation. This may lead some unfit patients to undergo shaping surgery, resulting in a poor prognosis. If the outcome of patients receiving MVP can be predicted through imaging examination before surgery, accurate treatment can be selected, which may further reduce the reoperation rate and improve the efficacy of MVP. Jia et al. and Wang et al. examined the efficacies of ultrasound and cardiac computed tomography angiography, respectively^26,27^. However, further studies and verification are needed to evaluate their effectiveness and standardize the scoring system.

PMBC, a classic treatment for rheumatic mitral stenosis, is recommended as a first-line treatment in international guidelines^7,8^. However, clinical trials supporting PMBC as a first-line treatment were conducted more than three decades ago in the 1990s. The advances in MVP and MVR in the 1990s are different from those today, and even the outcome of clinical trials in the 1990s revealed that the outcomes of PMBC were not significantly different from those of surgery^28,29^. PMBC is a minimally invasive surgery that is easy to perform in developing countries without surgical conditions. However, this does not provide credible evidence for PMBC being the preferred treatment for all patients described in the guidelines. Song et al. reported that open heart surgery has better long-term outcomes than PMBC^30^. Additionally, Coutinho et al. reported that after PMBC surgery, leaflet tearing was not common at the healthy commissure^14^. This increases the difficulty in undergoing MVP for patients who have undergone PMBC and subsequent secondary surgeries. In particular, young patients who initially undergo PMBC are at risk of requiring a redo-surgery, during which the option of MVP may be precluded owing to leaflet and commissure injury. If the patients prioritize MVP, a successful repair may prevent patients from undergoing valve surgery again for life. Based on the results of this study, composite MVP should be the preferred treatment option for patients who are eligible for this surgery. PMBC can be used for patients who cannot tolerate surgical intervention or those who are not suitable for composite MVP surgery.

This study has some limitations. First, this study included observational cohort studies in the analysis due to the lack of a sufficient number of RCTs. Second, MVP outcomes were associated with heterogeneity, which was further explained through subgroup analysis. Third, the annualized rates of outcomes were not provided, and the duration of follow-up periods varied among different studies. However, within each study, the follow-up time was comparable, thereby minimizing significant bias in the research findings. Finally, the duration among article publications was large and the sample size for the three groups was not uniform, which may lead to biased conclusions.

In this study, network meta-analysis revealed that the early mortality outcomes of MVP and PMBC were significantly better than those of MVR. There was no difference in the follow-up mortality and complications between MVP and PMBC. But the reoperation rate was much lower for MVP. Moreover, MVP had lower follow-up mortality and complication rates compared to MVR. This review also demonstrated that composite MVP is associated with significantly better outcomes than MVP with only commissurotomy. Composite MVP may serve as the preferred treatment option for rheumatic mitral valve disease, offering potentially improved long-term benefits relative to PMBC. Thus, the optimal surgical strategy should be individualized to achieve improved prognoses in patients with rheumatic mitral valve disease. Furthermore, additional clinical trials are necessary to confirm the findings of this study.

## Method

### Literature Search Strategy

This study followed the PRISMA (Preferred Reporting Items for Systematic Reviews and Meta-Analysis) guidelines and AMSTAR (Assessing the methodological quality of systematic reviews) guidelines for reporting systematic reviews^31,32^. Two independent reviewers conducted a comprehensive literature search and evaluated all relevant studies published in PubMed, Web of Science, and the Cochrane Central Registry. This study focused on retrospective and prospective cohort studies, as well as on RCTs, published in the English language comparing the efficacies of PMBC, MVP, and MVR. This review has been registered at the International Prospective Register of Systematic Reviews.

### Selection Criteria

This review selected studies published in the English language that compared the efficacies of PMBC, MVP, and MVR. Two reviewers independently searched the literature and evaluated all the articles. The inclusion criteria were as follows: 1) cohort studies or RCTs; 2) studies reporting baseline and postoperative results of patients with RHD undergoing PMBC, MVP, or MVR and evaluating at least one of the following outcomes: early mortality, follow-up mortality, and follow-up reoperation; 3) studies published in the English language.

The exclusion criteria were as follows: 1) studies with patients aged < 18 years; 2) studies that did not report the preoperative and postoperative data in the outcome measurements; 3) basic or animal studies; 4) studies whose full English texts were unavailable. The database search strategy is shown in Table S6.

### Data Extraction and Quality Assessment

All data, including study characteristics (e.g., publication year, author), patient demographics (e.g., age, gender, and Wilkins score), and outcomes, were extracted from article texts, tables, and fig. in different groups. The study characteristics for the network meta-analysis among PMBC, MVP, and MVR are shown in Tables S1 and S4. Besides, the early and late clinical results in studies among the three groups are shown in Table S5. Two investigators, working independently, reviewed every included article. When differences in opinion arose between the two reviewers, they were settled through discussion with a third reviewer. Details of appraisal and quality scoring are outlined in Table S2 according to the Newcastle-Ottawa Scale (NOS) for cohort studies. We used eight domains to assess the risks of bias, which included representativeness of exposed cohort, selection of nonexposed cohort, ascertainment of exposure, absence of outcome at start of study, comparability of cohorts, outcome assessment, length of follow-up, and adequacy of follow-up. We used five domains to assess the risks of bias for RCTs (Table S3), which included randomization process, deviations from intended interventions, missing outcome data, measurement of the outcome, and selection of the report result. All included studies were classified into a low, high, or unclear risk for each of bias.

### Outcomes

The assessed outcomes were the early mortality rate and follow-up mortality, reoperation, and complication rates in patients undergoing PMBC, MVP, or MVR. Early mortality refers to mortality within 30 days of surgery and in-hospital mortality. The complications encompassed bleeding, thrombosis, and severe mitral valve dysfunction during follow-up. Outcomes were analyzed across all postoperative follow-up periods.

### Statistical Analysis

This statistical analysis comprised the following five steps^33^: visualizing the network relationship, assessing consistency assumptions, illustrating comparative effectiveness with a network forest plot, ranking interventions based on cumulative rankings, and evaluating publication bias.

A random effects model was used for pairwise meta-analysis with ORs and 95% CIs for direct treatment comparisons. Heterogeneity was assessed using *I*^*2*^ (a statistical method to quantify heterogeneity) and *τ*^*2*^ (DerSimonian & Laird estimator). A network meta-analysis, which was performed using the frequentist method, was used to compare the efficacy and safety of PMBC, MVP, and MVR in patients with rheumatic mitral valve disease with ORs and 95% CIs based on a random effects model^34^. To establish a comparative hierarchy of procedural efficacy and safety, the “rankograms” with SUCRA probabilities were reported. SUCRA is regarded as a precise estimation for cumulative ranking probabilities^35^. Notably, a greater SUCRA value signifies that the corresponding surgical technique is ranked higher^36^. All statistical analyses were conducted using STATA MP 17.0 (Stata) and R 4.2.1.

## Supplementary information

Supplemental data

## Data Availability

All data have been reported in this network meta-analysis.

## References

[1] Coffey, S. et al. Global epidemiology of valvular heart disease. *Nat. Rev. Cardiol.* **18**, 853–864 (2021). 34172950 10.1038/s41569-021-00570-z

[2] Jiao, Y., Luo, T., Meng, X. & Wang, J. Decade-long mitral valve surgery trends and rheumatic heart disease: a review of mitral valve surgery in a large Chinese cardiovascular center. *Ann. Palliat. Med.* **11**, 1160–1169 (2022). 34894709 10.21037/apm-21-2005

[3] Pradegan, N. et al. Contemporary trends in surgical rheumatic valve disease in a Caribbean nation. *Int. J. Cardiol.* **328**, 215–217 (2021). 33309762 10.1016/j.ijcard.2020.12.023

[4] Hamsanathan, P. et al. A Review of Cardiac Surgical Procedures and Their Outcomes for Paediatric Rheumatic Heart Disease in Western Australia. *Heart Lung Circulation* **32**, 1398–1406 (2023). 37852820 10.1016/j.hlc.2023.08.012

[5] Rwebembera, J. et al. Clinical profile and outcomes of rheumatic heart disease patients undergoing surgical valve procedures in Uganda. *Glob. heart* **18**, 62 (2023). 38028964 10.5334/gh.1260 PMC10655755

[6] Zilla, P. et al. The Cape Town Declaration on access to cardiac surgery in the developing world. *J. Thorac. Cardiovasc. Surg.* **156**, 2206–2209 (2018). 30082076 10.1016/j.jtcvs.2018.06.002

[7] Heidenreich, P. A. et al. 2022 AHA/ACC/HFSA Guideline for the Management of Heart Failure: A Report of the American College of Cardiology/American Heart Association Joint Committee on Clinical Practice Guidelines. *Circulation* **145**, e895–e1032 (2022). 35363499 10.1161/CIR.0000000000001063

[8] Vahanian, A. et al. 2021 ESC/EACTS Guidelines for the management of valvular heart disease. *Eur. Heart J.*10.1093/eurheartj/ehab395 (2021). 10.1093/eurheartj/ehab395 34453165

[9] Bouleti, C. et al. Reinterventions after percutaneous mitral commissurotomy during long-term follow-up, up to 20 years: the role of repeat percutaneous mitral commissurotomy. *Eur. Heart J.* **34**, 1923–1930 (2013). 23514935 10.1093/eurheartj/eht097

[10] Chen, C. Y. et al. Bioprosthetic versus mechanical mitral valve replacements in patients with rheumatic heart disease. *J. Thorac. Cardiovasc. Surg.* **165**, 1050–1060.e1058 (2023). 33840468 10.1016/j.jtcvs.2021.03.033

[11] Kim, W. K. et al. Clinical outcomes in 1731 patients undergoing mitral valve surgery for rheumatic valve disease. *Heart* **104**, 841–848 (2018). 29146626 10.1136/heartjnl-2017-312249

[12] Fu, J. et al. Outcomes of mitral valve repair compared with replacement for patients with rheumatic heart disease. *J. Thorac. Cardiovasc. Surg.* **162**, 72–82 (2021). 32169372 10.1016/j.jtcvs.2020.01.053

[13] Dillon, J. et al. Comparative long-term results of mitral valve repair in adults with chronic rheumatic disease and degenerative disease: is repair for “burnt-out” rheumatic disease still inferior to repair for degenerative disease in the current era? *J. Thoracic Cardiovasc. Surg.* **149**, 771–777 (2015). 10.1016/j.jtcvs.2014.08.066 25308120

[14] Coutinho, G. F., Branco, C. F., Jorge, E., Correia, P. M. & Antunes, M. J. Mitral valve surgery after percutaneous mitral commissurotomy: is repair still feasible? *Eur. J. Cardio-Thorac. Surg. Off. J. Eur. Assoc. Cardio-Thorac. Surg.* **47**, e1–6 (2015). 10.1093/ejcts/ezu365 25694656

[15] Liao, Y. B., Wang, T. K. M., Wang, M. T. M., Ramanathan, T. & Wheeler, M. Meta-analysis of mitral valve repair versus replacement for rheumatic mitral valve disease. *Heart Lung Circul.* **31**, 705–710 (2022). 10.1016/j.hlc.2021.11.011 35120822

[16] Yasmin, F. et al. Comparative efficacy and safety of mitral valve repair versus mitral valve replacement in Rheumatic heart disease: a high-value care systematic review and meta-analysis. *Curr. Probl. Cardiol.* **49**, 102530, 10.1016/j.cpcardiol.2024.102530 (2024). 38518844 10.1016/j.cpcardiol.2024.102530

[17] Singh, A. D., Mian, A., Devasenapathy, N., Guyatt, G. & Karthikeyan, G. Percutaneous mitral commissurotomy versus surgical commissurotomy for rheumatic mitral stenosis: a systematic review and meta-analysis of randomised controlled trials. *Heart* **106**, 1094–1101 (2020). 31974210 10.1136/heartjnl-2019-315906

[18] Karthikeyan, G. et al. Mortality and morbidity in adults with rheumatic heart disease. *Jama* **332**, 133–140 (2024). 38837131 10.1001/jama.2024.8258 PMC11154374

[19] Li, X. et al. Comparison of mitral valve repair versus percutaneous mitral balloon commissurotomy for patients with rheumatic heart disease: a single-centre study. *Heart Lung Circul.* **33**, 1450–1456 (2024). 10.1016/j.hlc.2024.05.005 38955596

[20] Kumar, R. K. et al. Contemporary diagnosis and management of rheumatic heart disease: implications for closing the gap: a scientific statement from the American heart association. *Circulation* **142**, e337–e357 (2020). 33073615 10.1161/CIR.0000000000000921

[21] David, T. E. Commentary: Repair or replace rheumatic mitral valves? *J. Thorac. Cardiovasc. Surg.* **162**, 84–85 (2021). 32145908 10.1016/j.jtcvs.2020.02.025

[22] Wan, S., Wei, X. & Meng, X. Commentary: repairing the rheumatic mitral valve-know the enemy and know yourself!. *J. Thorac. Cardiovasc. Surg.* **164**, 72–73 (2022). 32928550 10.1016/j.jtcvs.2020.08.029

[23] Jiang, Y., Wang, C., Li, G. & Chen, S. Clinical outcomes following surgical mitral valve repair or replacement in patients with rheumatic heart disease: a meta-analysis. *Ann. Transl. Med.* **9**, 204 (2021). 33708831 10.21037/atm-20-3542 PMC7940942

[24] Fu, G. et al. Mitral valve surgery in patients with rheumatic heart disease: repair vs. replacement. *Front. Cardiovasc. Med.* **8**, 685746, 10.3389/fcvm.2021.685746 (2021). 34124209 10.3389/fcvm.2021.685746 PMC8193043

[25] De Bonis, M. & Carino, D. Commentary: rheumatic mitral valve disease: repair when you can, replace when you can’t! *J. Thorac. Cardiovasc. Surg.* **164**, 74–75 (2022). 33934895 10.1016/j.jtcvs.2020.08.062

[26] Jia, S. et al. Establishment and validation of a prediction model for the outcome of rheumatic mitral valve repair surgery based on transthoracic echocardiography. *Eur. Heart J.* **45**, 10.1093/eurheartj/ehae666.118 (2024).

[27] Wang, M. et al. Scoring model based on cardiac CT and clinical factors to predict early good mitral valve repair in rheumatic mitral disease. *Eur. Radiol.*10.1007/s00330-023-10470-0 (2024). 10.1007/s00330-023-10470-0 38252276

[28] Turi, Z. G. et al. Percutaneous balloon versus surgical closed commissurotomy for mitral stenosis. A prospective, randomized trial. *Circulation* **83**, 1179–1185 (1991). 2013139 10.1161/01.cir.83.4.1179

[29] Reyes, V. P. et al. Percutaneous balloon valvuloplasty compared with open surgical commissurotomy for mitral stenosis. *N. Engl. J. Med.* **331**, 961–967 (1994). 8084354 10.1056/NEJM199410133311501

[30] Song, J. K. et al. Long-term outcomes of percutaneous mitral balloon valvuloplasty versus open cardiac surgery. *J. Thorac. Cardiovasc. Surg.* **139**, 103–110 (2010). 19660411 10.1016/j.jtcvs.2009.04.022

[31] Page, M. J. et al. The PRISMA 2020 statement: an updated guideline for reporting systematic reviews. *Int J. Surg.* **88**, 105906, 10.1016/j.ijsu.2021.105906 (2021). 33789826 10.1016/j.ijsu.2021.105906

[32] Shea, B. J. et al. AMSTAR 2: a critical appraisal tool for systematic reviews that include randomised or non-randomised studies of healthcare interventions, or both. *BMJ* **358**, j4008 (2017). 28935701 10.1136/bmj.j4008 PMC5833365

[33] Shim, S., Yoon, B. H., Shin, I. S. & Bae, J. M. Network meta-analysis: application and practice using Stata. *Epidemiol. Health* **39**, e2017047 (2017). 29092392 10.4178/epih.e2017047 PMC5733388

[34] Higgins, J. P. et al. Consistency and inconsistency in network meta-analysis: concepts and models for multi-arm studies. *Res Synth. Methods* **3**, 98–110 (2012). 26062084 10.1002/jrsm.1044 PMC4433772

[35] Mbuagbaw, L. et al. Approaches to interpreting and choosing the best treatments in network meta-analyses. *Syst. Rev.* **6**, 79 (2017). 28403893 10.1186/s13643-017-0473-z PMC5389085

[36] Zhao, D. F. et al. Coronary artery bypass grafting with and without manipulation of the ascending aorta: a network meta-analysis. *J. Am. Coll. Cardiol.* **69**, 924–936 (2017). 28231944 10.1016/j.jacc.2016.11.071

