# Supplementary material for: Network meta-analysis to compare the efficacies of three surgical techniques in rheumatic mitral valve disease
Source: NPJ Cardiovasc Health. 2026 Feb 20;3:6. doi: 10.1038/s44325-026-00106-9 (PMC12923760; doi:10.1038/s44325-026-00106-9)
Supplement: Supplementary file 1 — Supplemental data [file 44325_2026_106_MOESM1_ESM.docx]

**Network meta-analysis to compare the efficacies of three surgical techniques in rheumatic mitral valve disease**

**Supplemental data**

**Table S1. Study characteristics for the network meta-analysis among PMBC, MVP and MVR.**

| **First Author** | **Year** | **Study Period** | **Country** | **N** | **PMBC** | **MVP** | **MVR** | **Study Type** | **Mainly lesion type** | **Mechanical valve, (%)** |
| --- | --- | --- | --- | --- | --- | --- | --- | --- | --- | --- |
| Promratpan | 2022 | 2010-2020 | Thailand | 264 | 164 | NR | 100 | Retro | Predominantly MS | 74 |
| Chen | 2022 | 2000-2013 | China | 5086 | NR | 489 | 4597 | Retro | Predominantly MS | NR |
| Fu | 2021 | 2011-2019 | China | 1644 | NR | 612 | 1032 | Retro | Mixed | 68 |
| Usta | 2021 | 1991-2012 | Turkey | 527 | 276 | NR | 251 | Retro | Predominantly MS | NR |
| Han | 2020 | 2013-2018 | China | 148 | 74 | 74 | NR | Retro | Predominantly MS | NR |
| Kim | 2018 | 1997-2015 | South Korea | 1731 | NR | 294 | 1437 | Retro | Mixed | 79 |
| Aslanabad | 2011 | 1999-2009 | Iran | 47 | 25 | NR | 22 | Retro | Predominantly MS | NR |
| Russel | 2017 | 2001-2013 | Australia | 1197 | NR | 119 | 1078 | Prosp | Mixed | NR |
| Kim | 2010 | 1997-2007 | South Korea | 540 | NR | 122 | 418 | Retro | Mixed | 100 |
| Wang | 2008 | 1997-2005 | China | 92 | NR | 33 | 59 | Retro | NR | 69 |
| Kuwaki | 2007 | 1981-2003 | Japan | 128 | NR | 47 | 81 | Retro | Predominantly MS | 81 |
| Talwar | 2007 | 1995-2005 | India | 369 | NR | 76 | 293 | Retro | Mixed | 100 |
| Song | 2007 | 1997-2005 | America | 92 | 48 | NR | 44 | Retro | Predominantly MS | NR |
| Kim | 2007 | 1998-2003 | South Korea | 91 | 32 | NR | 59 | Retro | Predominantly MS | NR |
| Ho | 2004 | 1992-2001 | Vietnam | 609 | NR | 201 | 408 | Retro | Mixed | 99 |
| Cardoso | 2002 | 1989-1994 | Brazil | 80 | 40 | 40 | NR | RCT | Predominantly MS | NR |
| Ismeso | 2000 | 1991-1997 | Italy | 313 | 111 | 82 | 120 | Retro | Predominantly MS | NR |
| Yau | 2000 | 1978-1995 | Canada | 573 | NR | 142 | 431 | Retro | Mixed | 63 |
| Ben | 1998 | 1987-1988 | Tunisia | 60 | 30 | 30 | NR | RCT | Predominantly MS | NR |
| Cotrufo | 1997 | 1981-1996 | Italy | 540 | NR | 300 | 240 | Retro | Predominantly MS | 100 |
| Reyes | 1994 | 1989-1989 | India | 60 | 30 | 30 | NR | RCT | Predominantly MS | NR |
| Cohen | 1993 | 1985-1990 | America | 164 | 64 | 40 | 60 | Retro | Predominantly MS | NR |
| Antunes | 1990 | 1981-1984 | South Africa | 916 | NR | 241 | 675 | Retro | Predominantly MR | 57 |

PMBC = percutaneous mitral valve balloon commissurotomy; MVP = mitral valvuloplasty; MVR = mitral valve replacement; Retro = retrospective observative study; Prosp = prospective observative study; RCT = randomized controlled trial; NR = not reported.

**Table S2. Quality assessment of the cohort studies included in the meta-analysis.**

| Studies, year | Representati-veness of exposed cohort | Selection of nonexposed cohort | Ascertainment of exposure | Absence of outcome at start of study | Comparability of cohorts | Outcome assessment | Length of follow-up | Adequacy of follow-up | NOS score |
| --- | --- | --- | --- | --- | --- | --- | --- | --- | --- |
| Ismeso, 2000 | 1 | 1 | 1 | 1 | 1 | 1 | 1 | 1 | 8 |
| Russel, 2017 | 1 | 1 | 1 | 1 | 1 | 1 | 0 | 0 | 6 |
| Cohen, 1993 | 1 | 1 | 1 | 1 | 1 | 1 | 1 | 1 | 8 |
| Usta, 2021 | 1 | 1 | 1 | 1 | 1 | 1 | 1 | 1 | 8 |
| Han, 2020 | 1 | 1 | 1 | 1 | 1 | 1 | 1 | 1 | 8 |
| Chen, 2022 | 1 | 1 | 1 | 1 | 1 | 1 | 1 | 1 | 8 |
| Fu, 2021 | 1 | 1 | 1 | 1 | 1 | 1 | 1 | 1 | 8 |
| Kim, 2018 | 1 | 1 | 1 | 1 | 1 | 1 | 1 | 1 | 8 |
| Kim, 2010 | 1 | 1 | 1 | 1 | 1 | 1 | 1 | 1 | 8 |
| Wang, 2008 | 0 | 0 | 1 | 1 | 1 | 1 | 1 | 1 | 6 |
| Kuwaki, 2007 | 1 | 1 | 1 | 1 | 1 | 1 | 1 | 1 | 8 |
| Talwar, 2007 | 1 | 1 | 1 | 1 | 1 | 1 | 1 | 1 | 8 |
| Ho, 2004 | 1 | 1 | 1 | 1 | 1 | 1 | 1 | 1 | 8 |
| Yau, 2000 | 1 | 1 | 1 | 1 | 1 | 1 | 1 | 1 | 8 |
| Cotrufo, 1997 | 1 | 1 | 1 | 1 | 1 | 1 | 1 | 1 | 8 |
| Antunes, 1990 | 1 | 0 | 1 | 1 | 1 | 1 | 1 | 1 | 7 |
| Promratpan, 2022 | 1 | 1 | 1 | 1 | 1 | 1 | 1 | 1 | 8 |
| Aslanabad, 2011 | 1 | 1 | 1 | 1 | 1 | 1 | 1 | 1 | 8 |
| Song, 2007 | 1 | 1 | 1 | 1 | 1 | 1 | 1 | 1 | 8 |
| Kim, 2007 | 1 | 1 | 1 | 1 | 1 | 1 | 1 | 1 | 8 |

NOS=Newcastle-Ottawa Scale

**Table S3. Quality assessment of the randomized control trials in the meta-analysis.**

| Studies, year | Randomization process | Deviations from intended interventions | Missing outcome data | Measurement of the outcome | Selection of the reported result |
| --- | --- | --- | --- | --- | --- |
| Cardoso, 2002 | - | + | + | + | + |
| Ben, 1998 | ? | + | + | + | + |
| Reyes, 1994 | ? | + | + | + | + |

Risk of bias graph: the distribution of risk of bias judgments (Yes, Green; No, Red; Unclear, Yellow) for each study.

**Table S4. Characteristics for included studies in the meta-analysis among PMBC, MVP, and MVR.**

| First author, year | Groups | N | Age, year | F, (%) | Wilkins score | MVA, cm^2^ | NYHA III-IV | AF, (%) | Follow-  up, (m) | Comments (detailed techniques of valvuloplasty) |
| --- | --- | --- | --- | --- | --- | --- | --- | --- | --- | --- |
| PMBC/MVP/MVR | | | | | | | | | | |
| Ismeso, 2000 | PMBC | 111 | 46.5 ± 13.8 | 77 | NR | NR | NR | 34.2 | 37.7 | Valvuloplasty* (commissurotomy and/or papillarotomy) |
|  | MVP | 82 | 48.9 ± 10.0 | 87 | NR | NR | NR | 52.4 | 49.9 |  |
|  | MVR | 120 | 52.0 ± 18.0 | 74 | NR | NR | NR | 53.3 | 45.1 |  |
| Cohen, 1993 | PMBC | 64 | 50 ± 12 | 83 | NR | NR | NR | 58.0 | 30 | Valvuloplasty* （commissurotomy） |
|  | MVP | 40 | 53 ± 12 | 98 | NR | NR | NR | 60.0 | 45 |  |
|  | MVR | 60 | 55 ± 12 | 88 | NR | NR | NR | 73.0 | 34 |  |
| PMBC/MVP | | | | | | | | | | |
| Han, 2020 | PMBC | 74 | 46.95 ± 12.50 | 71.6 | NR | 0.97 ± 0.24 | 35.1 | 47.3 | 43.72 | Valvuloplasty (ring annuloplasty, leaflet thinning, commissurotomy, and/or release of subvalvular apparatus) |
|  | MVP | 74 | 47.55 ± 11.91 | 73 | NR | 1.05 ± 0.32 | 40.5 | 55.4 | 44.5 |  |
| Cardoso, 2002 | PMBC | 40 | 32 ± 9 | 92.5 | NR | 1.04 ± 0.23 | 87.5 | 2.5 | 24 | Valvuloplasty* (commissurotomy and/or papillarotomy) |
|  | MVP | 40 | 33 ± 8 | 87.5 | NR | 0.96 ± 0.2 | 92.5 | 7.5 | 24 |  |
| Ben, 1998 | PMBC | 30 | 29 ± 12 | 77 | 6 ± 1 | 0.9 ± 0.2 | 90 | NR | 84 | Valvuloplasty* (commissurotomy and/or papillarotomy) |
|  | MVP | 30 | 27 ± 9 | 70 | 6 ± 1 | 0.9 ± 0.2 | 87 | NR | 84 |  |
| Reyes, 1994 | PMBC | 30 | 30 ± 9 | 93 | 6.7 ± 1.3 | 0.9 ± 0.3 | 50 | NR | 36 | Valvuloplasty* (commissurotomy and/or release of subvalvular apparatus) |
|  | MVP | 30 | 31 ± 9 | 63 | 7 ± 1.1 | 0.9 ± 0.3 | 63.3 | NR | 36 |  |
| MVP/MVR | | | | | | | | | | |
| Chen, 2022 | MVP | 489 | 56.5 ± 14.4 | 51.7 | NR | NR | NR | 55.6 | 68.4 | Valvuloplasty (not detailed) |
|  | MVR | 4597 | 58.1 ± 12.7 | 43.0 | NR | NR | NR | 67.9 | 61.2 |  |
| Fu, 2021 | MVP | 612 | 53.4 ± 11.3 | 73 | NR | NR | NR | 63.9 | 49.4 | Valvuloplasty (ring annuloplasty, leaflet thinning, commissurotomy, and/or release of subvalvular apparatus) |
|  | MVR | 1032 | 56.1 ± 9.8 | 70.2 | NR | NR | NR | 77.0 | 49.4 |  |
| Kim, 2018 | MVP | 294 | 43.9 ± 13.9 | 76.2 | NR | NR | NR | 56.8 | 130.9 | Valvuloplasty (ring annuloplasty, commissurotomy, and/or release of subvalvular apparatus) |
|  | MVR | 1437 | 54 ± 11 | 67.2 | NR | NR | NR | 75.2 | 130.9 |  |
| Russel, 2017 | MVP | 119 | 57.3 ± 16.9 | 58.0 | NR | NR | 42.9 | 26.1 | NR | Valvuloplasty (not detailed) |
|  | MVR | 1078 | 62.0 ± 10.4 | 71.3 | NR | NR | 58.4 | 48.9 | NR |  |
| Kim, 2010 | MVP | 122 | 41.7 ± 13.2 | 77.9 | NR | 2.7 ± 1.2 | NR | 73 | 71.8 | Valvuloplasty (ring annuloplasty, commissurotomy, and/or release of subvalvular apparatus) |
|  | MVR | 418 | 51.0 ± 10.1 | 62.4 | NR | 1.3 ± 0.8 | NR | 94.5 | 71.8 |  |
| Wang, 2008 | MVP | 33 | 49.7 ± 13.2 | 63.7 | NR | NR | 84.8 | 93.9 | 33.6 | Valvuloplasty (Carpentier’s techniques) |
|  | MVR | 59 | 58.1 ± 11.2 | 66.1 | NR | NR | 94.9 | 96.6 | 37.2 |  |
| Kuwaki, 2007 | MVP | 47 | 48 ± 10 | 70 | NR | NR | NR | NR | 109.2 | Valvuloplasty (ring annuloplasty and/or commissurotomy), with aortic valve replacement. |
|  | MVR | 81 | 53 ± 8 | 58 | NR | NR | NR | NR | 109.2 |  |
| Talwar, 2007 | MVP | 76 | 30.3 ± 10.4 | 30.3 | NR | NR | 75.0 | 48.7 | 69 | Valvuloplasty (ring annuloplasty, leaflet thinning, commissurotomy, and/or release of subvalvular apparatus) |
|  | MVR | 293 | 32.5 ± 10.7 | 27 | NR | NR | 72.3 | 45.4 | 52 |  |
| Ho, 2004 | MVP | 201 | 32.2 ± 10.4 | 46.3 | NR | NR | 19.4 | 36.8 | 62 | Valvuloplasty (Carpentier’s techniques); With aortic valve replacement. |
|  | MVR | 408 | 38.7 ± 8.6 | 44.4 | NR | NR | 15.7 | 60.3 | 37.5 |  |
| Yau, 2000 | MVP | 142 | 42 ± 1.1 | 85 | NR | NR | NR | 31.7 | 78 | Valvuloplasty (not detailed) |
|  | MVR | 431 | 58 ± 0.8 | 79 | NR | NR | NR | 64.3 | 65 |  |
| Cotrufo, 1997 | MVP | 300 | 43 ± 12 | 88.7 | NR | NR | NR | NR | 92 | Valvuloplasty （simple commissurotomy） |
|  | MVR | 240 | 50 ± 12 | 72.5 | NR | NR | NR | NR | 53 |  |
| Antunes, 1990 | MVP | 241 | 21.5 ± 11.8 | NR | NR | NR | NR | NR | 44.4 | Valvuloplasty (Carpentier’s techniques) |
|  | MVR | 675 | 26.95 ± 13.87 | NR | NR | NR | NR | NR | 54 |  |
| PMBC/MVR | | | | | | | | | | |
| Promratpan, 2022 | PMBC | 164 | 47.38 ± 13.38 | 79.9 | 8.10 ± 1.56 | 0.94 ± 0.27 | NR | 53 | 62.5 |  |
|  | MVR | 100 | 53.04 ± 11.69 | 82.0 | 9.47 ± 2.01 | 0.85 ± 0.32 | NR | 94 | 57 |  |
| Usta, 2021 | PMBC | 276 | 40.88 ± 11.56 | 84.8 | NR | NR | 54 | 33.3 | 56.4 |  |
|  | MVR | 251 | 51.49 ± 11.51 | 68.9 | NR | NR | 49.8 | 58.6 | 65.4 |  |
| Aslanabad, 2011 | PMBC | 25 | 40.7 ± 11 | 76 | 9.56 ± 1.47 | 0.97 ± 0.1 | NR | 32 | 41 | With previous PMBC |
|  | MVR | 22 | 47 ± 10.3 | 69 | 10.59±2.48 | 1.15 ± 0.34 | NR | 63.3 | 63 |  |
| Song, 2007 | PMBC | 48 | 44 ± 11 | 85 | NR | 0.9 ± 0.2 | NR | 54 | 57 | With severe tricuspid regurgitation |
|  | MVR | 44 | 54 ± 12 | 75 | NR | 1.0 ± 0.3 | NR | 86 | 57 |  |
| Kim, 2007 | PMBC | 32 | 42 ± 11 | 94 | 8.4 ± 1.0 | 1.03 ± 0.2 | 48 | 29 | 59 | With previous PMBC |
|  | MVR | 59 | 50 ± 10 | 70 | 9.2 ± 2.3 | 1.42 ± 1.82 | 61 | 55 | 98 |  |

* Refers to simple commissurotomy; PMBC = percutaneous mitral valve balloon commissurotomy; MVP = mitral valvuloplasty; MVR = mitral valve replacement; F = female; MVA = mitral valve orifice area; NYHA = New York Heart Association; AF = atrial fibrillation; NR = not reported.

**Table S5. Early and late clinical results reported in studies included in the meta-analysis among PMBC, MVP, and MVR.**

| First author, year | Groups | N | Follow-up, (m) | Follow-up, (y) | Early mortality (n) | Follow-up mortality (n) | Follow-up reoperation (n) | Follow-up complications (n) |
| --- | --- | --- | --- | --- | --- | --- | --- | --- |
| PMBC/MVP/MVR | | | | | | | | |
| Ismeso, 2000 | PMBC | 111 | 37.7 | 3.1 | 0 | 2 | 4 | 2 |
|  | MVP | 82 | 49.9 | 4.2 | 0 | 1 | 2 | 1 |
|  | MVR | 120 | 45.1 | 3.8 | 2 | 4 | 2 | 8 |
| Cohen, 1993 | PMBC | 64 | 30 | 2.5 | 1 | 9 | 17 | NR |
|  | MVP | 40 | 45 | 3.8 | 1 | 2 | 6 | NR |
|  | MVR | 60 | 34 | 2.8 | 2 | 5 | 2 | NR |
| PMBC/MVP | | | | | | | | |
| Han, 2020 | PMBC | 74 | 43.72 | 3.6 | 0 | 1 | 11 | NR |
|  | MVP | 74 | 44.5 | 3.7 | 0 | 1 | 0 | NR |
| Cardoso, 2002 | PMBC | 40 | 24 | 2 | 0 | 0 | 0 | NR |
|  | MVP | 40 | 24 | 2 | 0 | 0 | 0 | NR |
| Ben, 1998 | PMBC | 30 | 84 | 7 | 0 | 0 | 2 | 6 |
|  | MVP | 30 | 84 | 7 | 0 | 0 | 2 | 6 |
| Reyes, 1994 | PMBC | 30 | 36 | 3 | 0 | 1 | 3 | 5 |
|  | MVP | 30 | 36 | 3 | 0 | 0 | 4 | 5 |
| MVP/MVR | | | | | | | | |
| Chen, 2022 | MVP | 489 | 68.4 | 5.7 | 33 | 162 | 35 | 125 |
|  | MVR | 4597 | 61.2 | 5.1 | 309 | 1481 | 85 | 1172 |
| Fu, 2021 | MVP | 612 | 49.4 | 4.1 | 3 | 12 | 18 | NR |
|  | MVR | 1032 | 49.4 | 4.1 | 36 | 85 | 14 | NR |
| Kim, 2018 | MVP | 294 | 130.9 | 10.9 | 5 | 26 | 18 | 18 |
|  | MVR | 1437 | 130.9 | 10.9 | 69 | 183 | 34 | 206 |
| Russel, 2017 | MVP | 119 | NR | NR | 5 | NR | NR | NR |
|  | MVR | 1078 | NR | NR | 41 | NR | NR | NR |
| Kim, 2010 | MVP | 122 | 71.8 | 6.0 | 2 | NR | 3 | NR |
|  | MVR | 418 | 71.8 | 6.0 | 4 | NR | 11 | NR |
| Wang, 2008 | MVP | 33 | 33.6 | 2.8 | 1 | 4 | 2 | 2 |
|  | MVR | 59 | 37.2 | 3.1 | 4 | 6 | 0 | 12 |
| Kuwaki, 2007 | MVP | 47 | 109.2 | 9.1 | 2 | 6 | 21 | 5 |
|  | MVR | 81 | 109.2 | 9.1 | 3 | 19 | 12 | 10 |
| Talwar, 2007 | MVP | 76 | 69 | 5.8 | 4 | 5 | 7 | 37 |
|  | MVR | 293 | 52 | 4.3 | 25 | 26 | 1 | 210 |
| Ho, 2004 | MVP | 201 | 62 | 5.2 | 3 | 3 | 7 | 7 |
|  | MVR | 408 | 37.5 | 3.1 | 3 | 5 | 4 | 20 |
| Yau, 2000 | MVP | 142 | 78 | 6.5 | 1 | NR | NR | NR |
|  | MVR | 431 | 65 | 5.4 | 23 | NR | NR | NR |
| Cotrufo, 1997 | MVP | 300 | 92 | 7.7 | 6 | 3 | 27 | NR |
|  | MVR | 240 | 53 | 4.4 | 5 | 7 | 4 | NR |
| Antunes, 1990 | MVP | 241 | 44.4 | 3.7 | 8 | 15 | 25 | 35 |
|  | MVR | 675 | 54 | 4.5 | 47 | 111 | 55 | 132 |
| PMBC/MVR | | | | | | | | |
| Promratpan, 2022 | PMBC | 164 | 62.5 | 5.2 | 1 | 28 | 30 | 14 |
|  | MVR | 100 | 57 | 4.8 | 4 | 15 | 0 | 12 |
| Usta, 2021 | PMBC | 276 | 56.4 | 4.7 | 0 | 0 | 45 | 5 |
|  | MVR | 251 | 65.4 | 5.5 | 5 | 10 | 6 | 5 |
| Aslanabad, 2011 | PMBC | 25 | 41 | 3.4 | 0 | 1 | 8 | NR |
|  | MVR | 22 | 63 | 5.3 | 3 | 3 | 4 | NR |
| Song, 2007 | PMBC | 48 | 57 | 4.8 | 0 | 2 | 5 | NR |
|  | MVR | 44 | 57 | 4.8 | 0 | 2 | 0 | NR |
| Kim, 2007 | PMBC | 32 | 59 | 4.9 | 0 | 0 | 10 | NR |
|  | MVR | 59 | 98 | 8.2 | 0 | 0 | 0 | NR |

PMBC = percutaneous mitral valve balloon commissurotomy; MVP = mitral valvuloplasty; MVR = mitral valve replacement; NR = not reported.

**Table S6. Search strategy for databases**

| **Search keywords** | **Search database** |
| --- | --- |
| ((rheumatic heart disease) OR (rheumatic mitral valve disease) OR (RHD) OR (mitral stenosis)) AND ((percutaneous mitral balloon commissurotomy) OR(Percutaneous Balloon Mitral Valvuloplasty）OR (Percutaneous Transvenous Mitral Commissurotomy) OR (percutaneous mitral commissurotomy) OR (PMBC) OR (open surgical commissurotomy) OR (mitral valve repair) OR (mitral valve reconstruction) OR (mitral valve surgery) OR (MVP) OR (mitral valvuloplasty) OR (mitral valve replacement))AND ((mortality) OR (reoperation)) Filters: from 1950/1/1 - 2023/10/8 | PubMed, Cochrane Central Registry, Web of science |

**References:**

1. Antunes MJ. Mitral valvuloplasty, a better alternative. Comparative study between valve reconstruction and replacement for rheumatic mitral valve disease. Eur J Cardiothorac Surg. 1990;4(5):257-262; discussion 263-254.

2. Aslanabadi N, Golmohammadi A, Sohrabi B, Kazemi B. Repeat percutaneous balloon mitral valvotomy vs. mitral valve replacement in patients with restenosis after previous balloon mitral valvotomy and unfavorable valve characteristics. Clin Cardiol. 2011;34(6):401-406.

3. Ben Farhat M, Ayari M, Maatouk F, Betbout F, Gamra H, Jarra M, et al. Percutaneous balloon versus surgical closed and open mitral commissurotomy: seven-year follow-up results of a randomized trial. Circulation. 1998;97(3):245-250.

4. Cardoso LF, Grinberg M, Rati MA, Pomerantzeff PM, Medeiros CC, Tarasoutchi F, et al. Comparison between percutaneous balloon valvuloplasty and open commissurotomy for mitral stenosis. A prospective and randomized study. Cardiology. 2002;98(4):186-190.

5. Chen SW, Chen CY, Chien-Chia Wu V, Chou AH, Cheng YT, Chang SH, et al. Mitral valve repair versus replacement in patients with rheumatic heart disease. J Thorac Cardiovasc Surg. 2022;164(1):57-67.e11.

6. Cohen JM, Glower DD, Harrison JK, Bashore TM, White WD, Smith LR, et al. Comparison of balloon valvuloplasty with operative treatment for mitral stenosis. Ann Thorac Surg. 1993;56(6):1254-1262.

7. Cotrufo M, Renzulli A, Vitale N, Nappi G, De Feo M, Ismeno G, et al. Long-term follow-up of open commissurotomy versus bileaflet valve replacement for rheumatic mitral stenosis. Eur J Cardiothorac Surg. 1997;12(3):335-339; discussion 339-340.

8. Fu J, Li Y, Zhang H, Han J, Jiao Y, Du J, et al. Outcomes of mitral valve repair compared with replacement for patients with rheumatic heart disease. J Thorac Cardiovasc Surg. 2021;162(1):72-82.e77.

9. Han J, Tian B, Wu F, Jiao Y, Pang S, Xu J, et al. Surgical rheumatic mitral valve repair compared with percutaneous balloon mitral valvuloplasty in mitral stenosis in current era: a propensity score matching study. J Thorac Dis. 2020;12(11):6752-6760.

10. Ho HQ, Nguyen VP, Phan KP, Pham NV. Mitral valve repair with aortic valve replacement in rheumatic heart disease. Asian Cardiovasc Thorac Ann. 2004;12(4):341-345.

11. Ismeno G, Renzulli A, De Feo M, Della Corte A, Mauro C, Romano G, et al. Surgery of rheumatic mitral stenosis: comparison of different techniques. Acta Cardiol. 2001;56(3):155-161.

12. Kim JB, Ha JW, Kim JS, Shim WH, Kang SM, Ko YG, et al. Comparison of long-term outcome after mitral valve replacement or repeated balloon mitral valvotomy in patients with restenosis after previous balloon valvotomy. Am J Cardiol. 2007;99(11):1571-1574.

13. Kim JB, Kim HJ, Moon DH, Jung SH, Choo SJ, Chung CH, et al. Long-term outcomes after surgery for rheumatic mitral valve disease: valve repair versus mechanical valve replacement. Eur J Cardiothorac Surg. 2010;37(5):1039-1046.

14. Kim WK, Kim HJ, Kim JB, Jung SH, Choo SJ, Chung CH, et al. Clinical outcomes in 1731 patients undergoing mitral valve surgery for rheumatic valve disease. Heart. 2018;104(10):841-848.

15. Kuwaki K, Kawaharada N, Morishita K, Koyanagi T, Osawa H, Maeda T, et al. Mitral valve repair versus replacement in simultaneous mitral and aortic valve surgery for rheumatic disease. Ann Thorac Surg. 2007;83(2):558-563.

16. Promratpan W, Theerasuwipakorn N, Lertsuwunseri V, Srimahachota S. Long-term outcomes of severe rheumatic mitral stenosis after undergoing percutaneous mitral commissurotomy and mitral valve replacement: A 10-year experience. J Cardiovasc Thorac Res. 2022;14(2):101-107.

17. Reyes VP, Raju BS, Wynne J, Stephenson LW, Raju R, Fromm BS, et al. Percutaneous balloon valvuloplasty compared with open surgical commissurotomy for mitral stenosis. N Engl J Med. 1994;331(15):961-967.

18. Russell EA, Walsh WF, Reid CM, Tran L, Brown A, Bennetts JS, Baker RA, Tam R, Maguire GP. Outcomes after mitral valve surgery for rheumatic heart disease. Heart Asia. 2017 Jun 19;9(2):e010916

19. Song H, Kang DH, Kim JH, Park KM, Song JM, Choi KJ, et al. Percutaneous mitral valvuloplasty versus surgical treatment in mitral stenosis with severe tricuspid regurgitation. Circulation. 2007;116(11 Suppl):I246-250.

20. Talwar S, Mathur A, Choudhary SK, Singh R, Kumar AS. Aortic valve replacement with mitral valve repair compared with combined aortic and mitral valve replacement. Ann Thorac Surg. 2007;84(4):1219-1225.

21. Usta E, Erdim R, Görmez S, Dogan A, Ezelsoy M, Kahraman S, et al. Comparison of early and long-term follow-up results of percutaneous mitral balloon valvuloplasty and mitral valve replacement. Rev Assoc Med Bras (1992). 2021;67(1):58-63.

22. Wang YC, Tsai FC, Chu JJ, Lin PJ. Midterm outcomes of rheumatic mitral repair versus replacement. Int Heart J. 2008;49(5):565-576.

23. Yau TM, El-Ghoneimi YA, Armstrong S, Ivanov J, David TE. Mitral valve repair and replacement for rheumatic disease. J Thorac Cardiovasc Surg. 2000;119(1):53-60


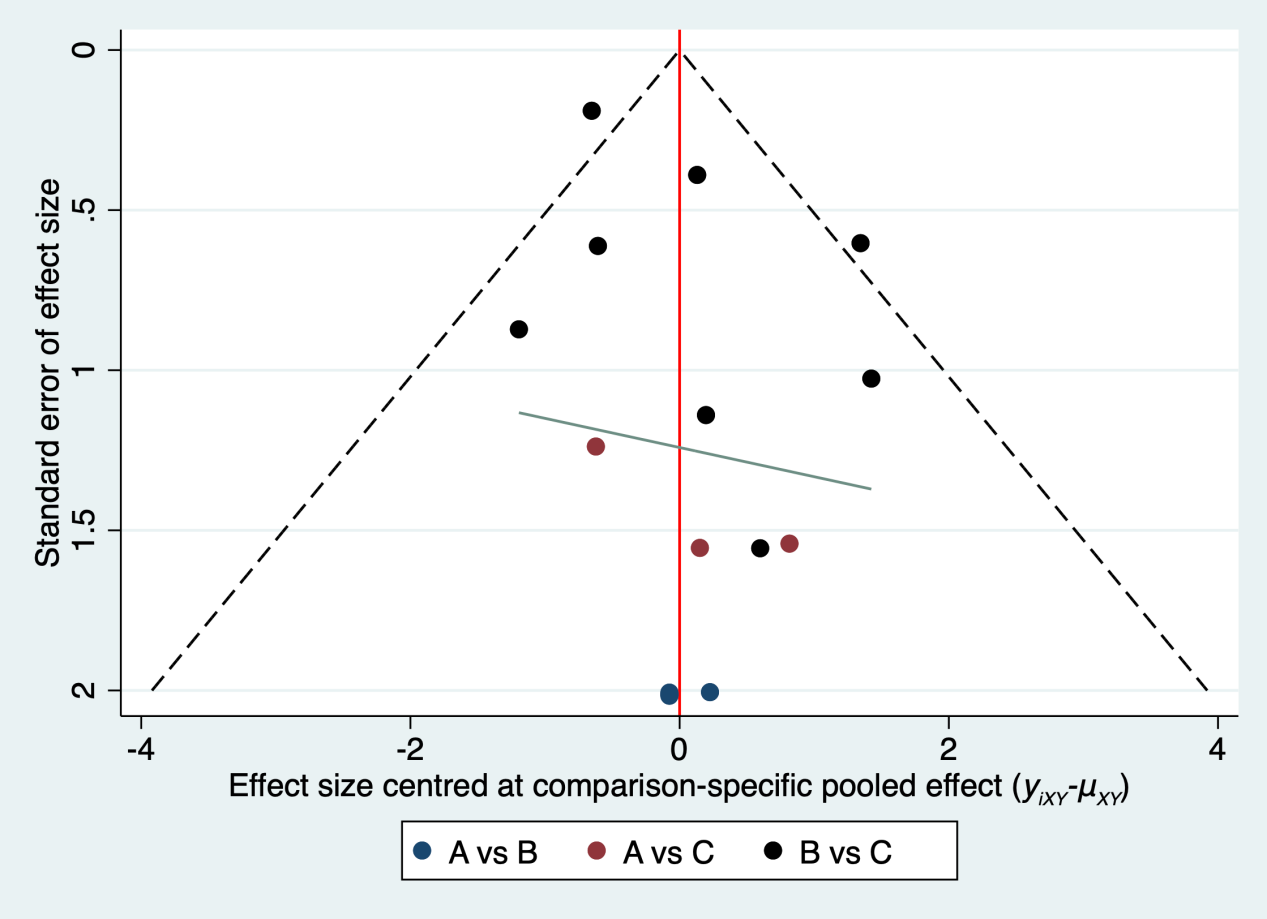


Figure S1. Comparison-adjusted funnel plot for early mortality. (A, PMBC; B, MVP; C, MVR)


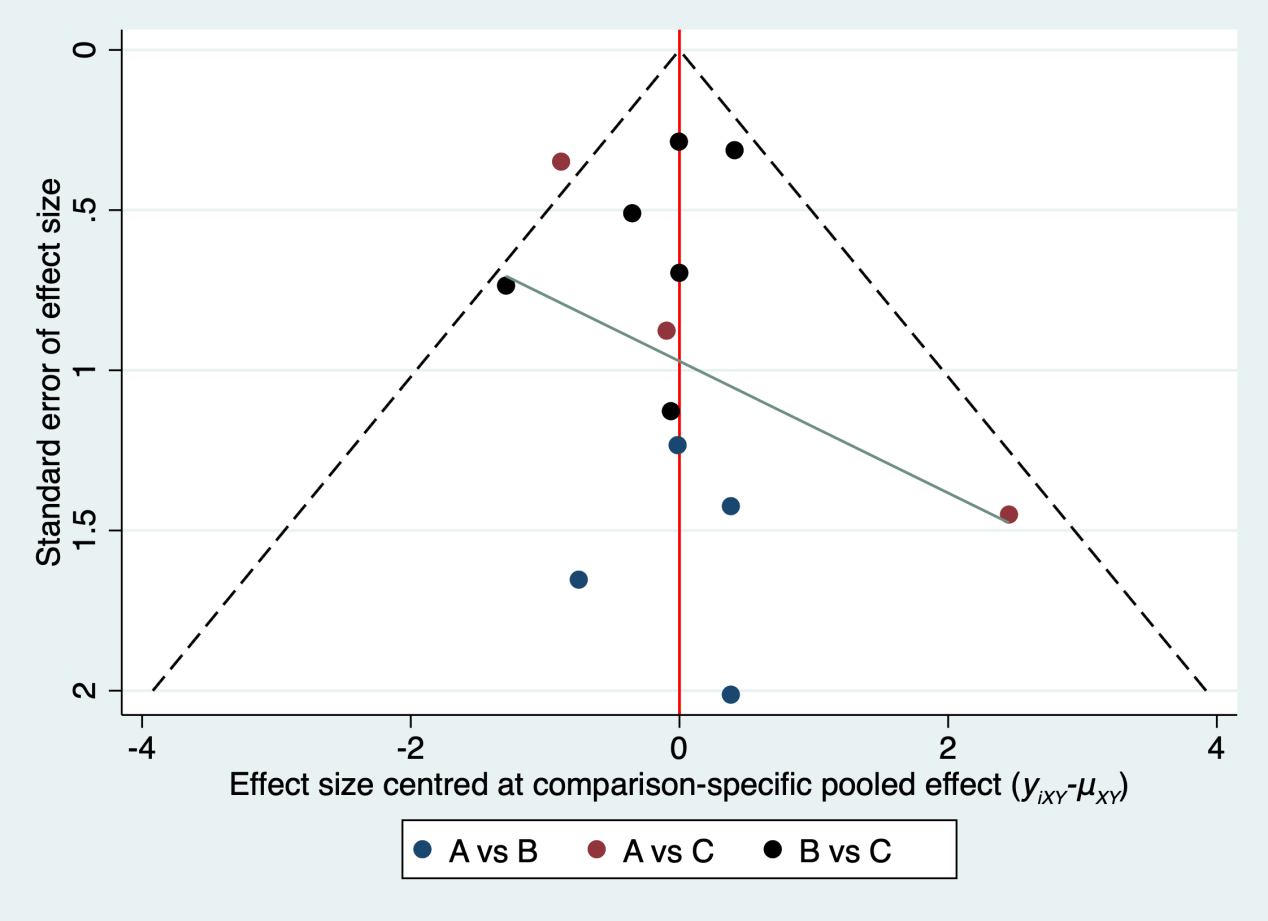


Figure S2. Comparison-adjusted funnel plot for follow-up mortality. (A, PMBC; B, MVP; C, MVR)


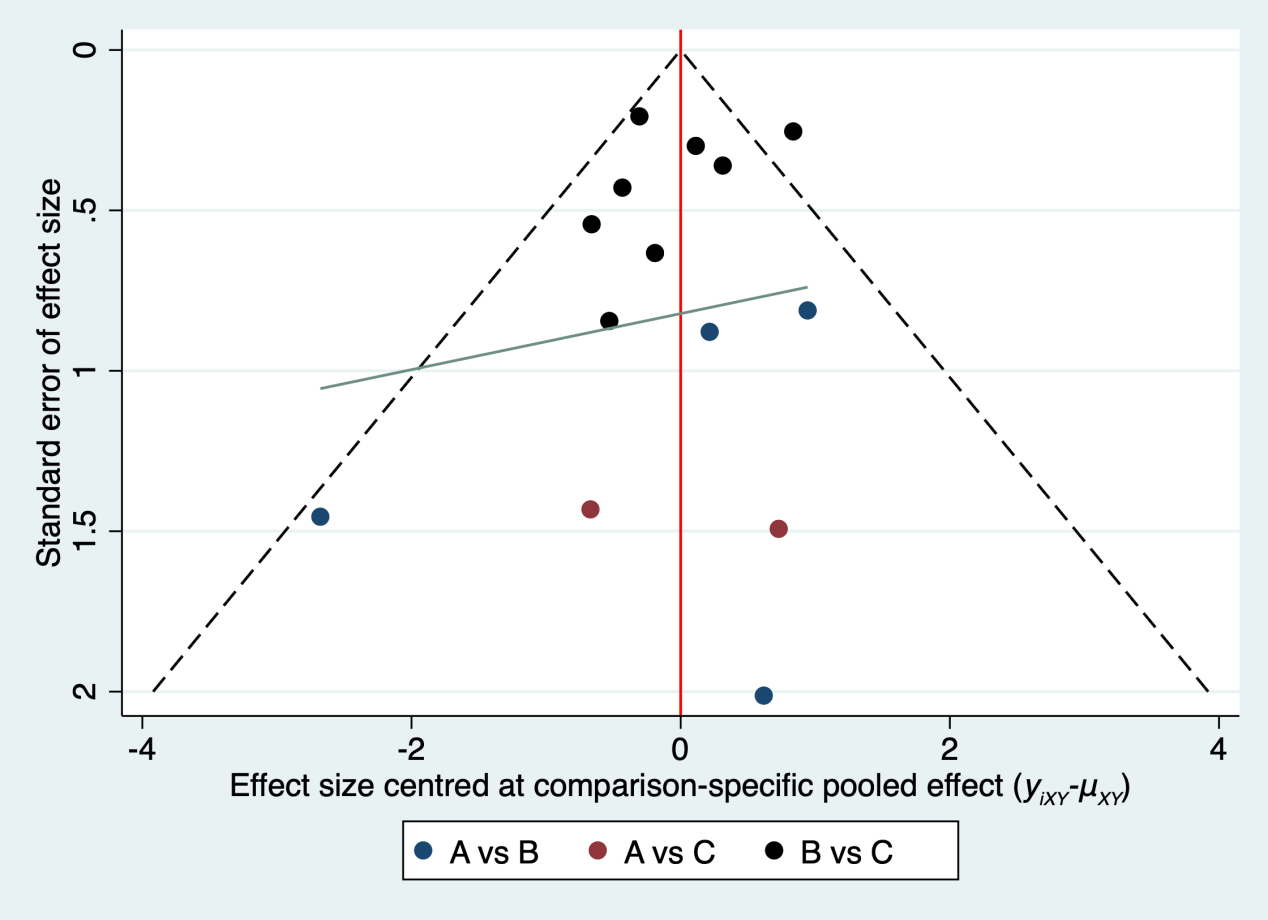


Figure S3. Comparison-adjusted funnel plot for follow-up reoperation. (A, PMBC; B, MVP; C, MVR)


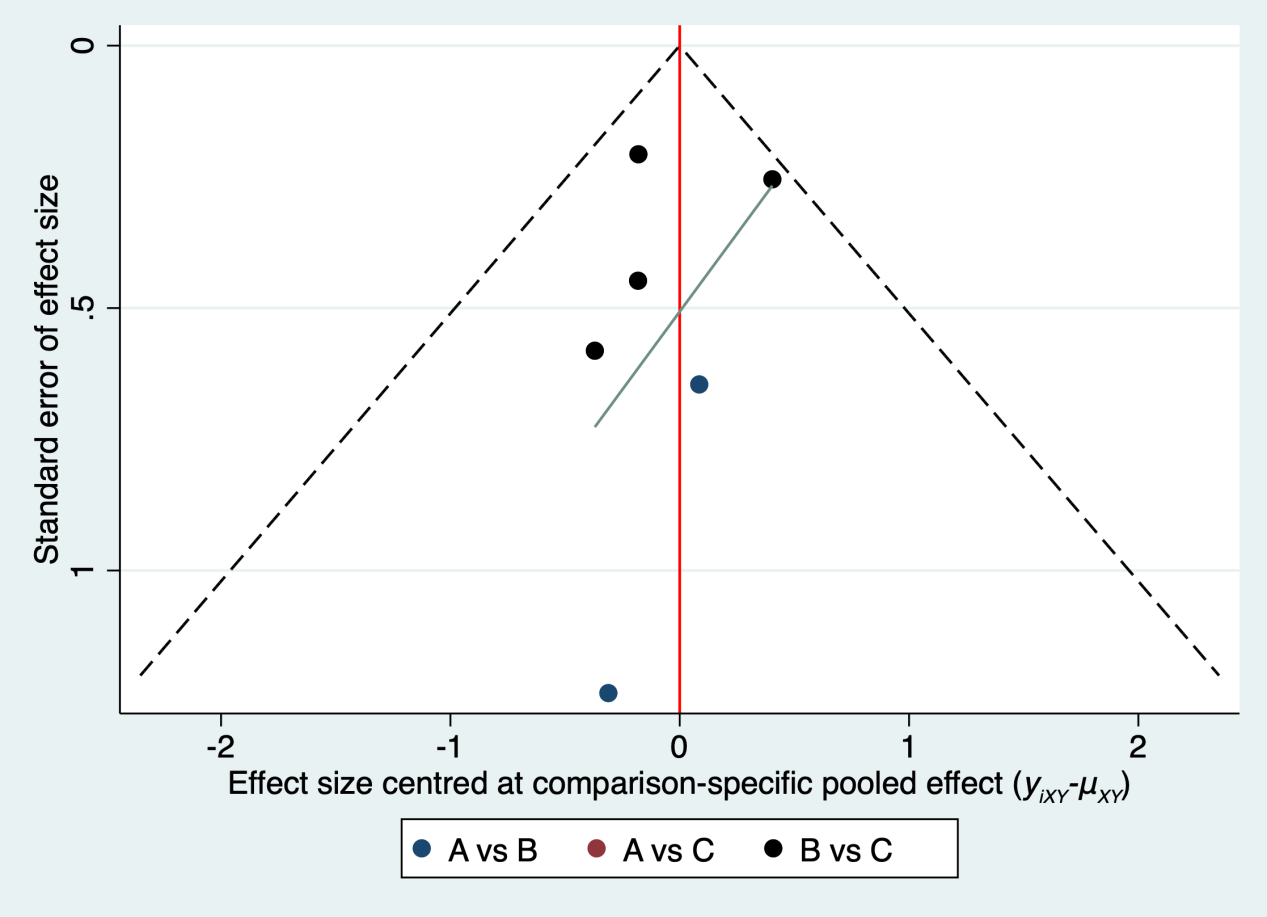


Figure S4. Comparison-adjusted funnel plot for follow-up complications. (A, PMBC; B, MVP; C, MVR)


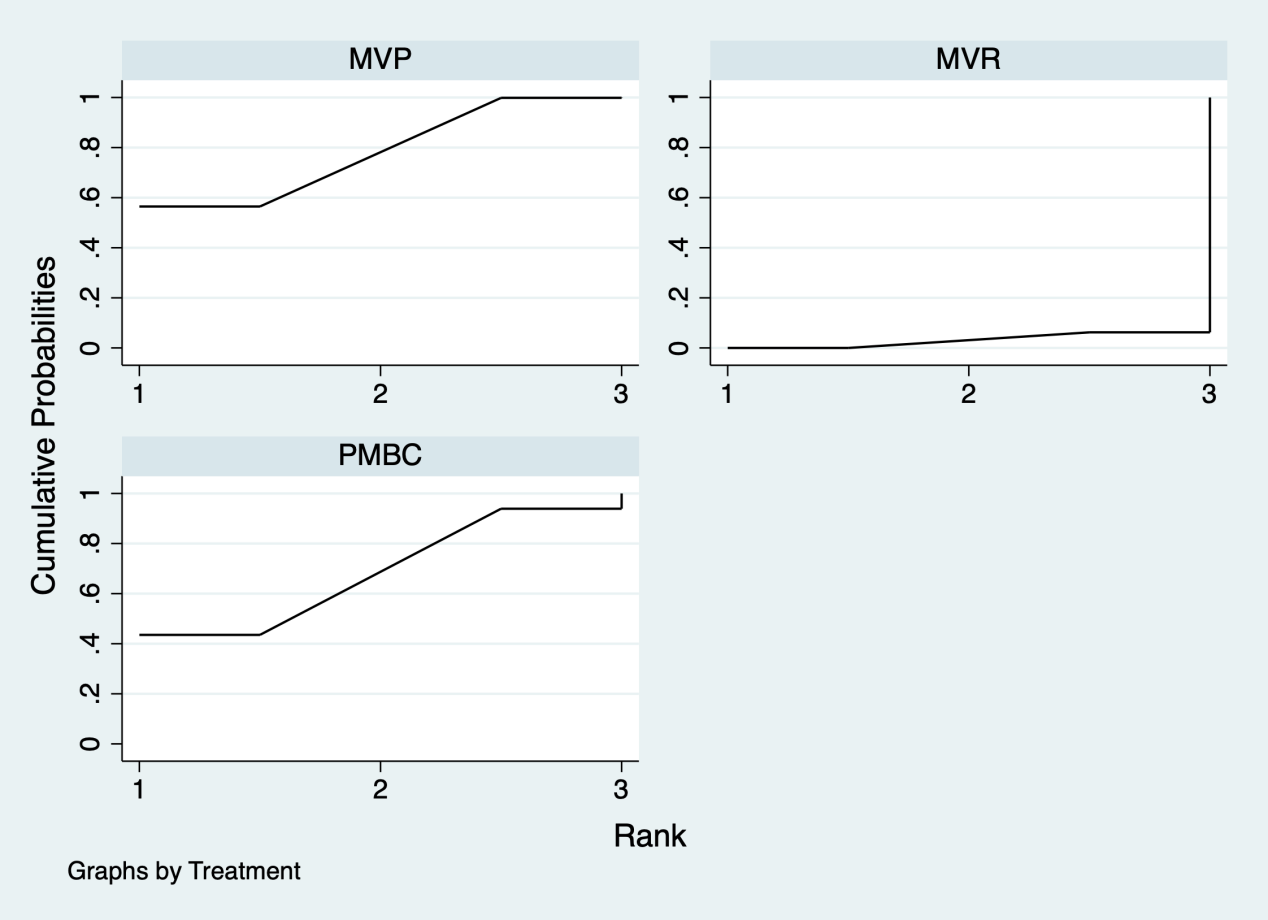


Figure S5. Plots of cumulative ranking probability on early mortality. (SUCRA)


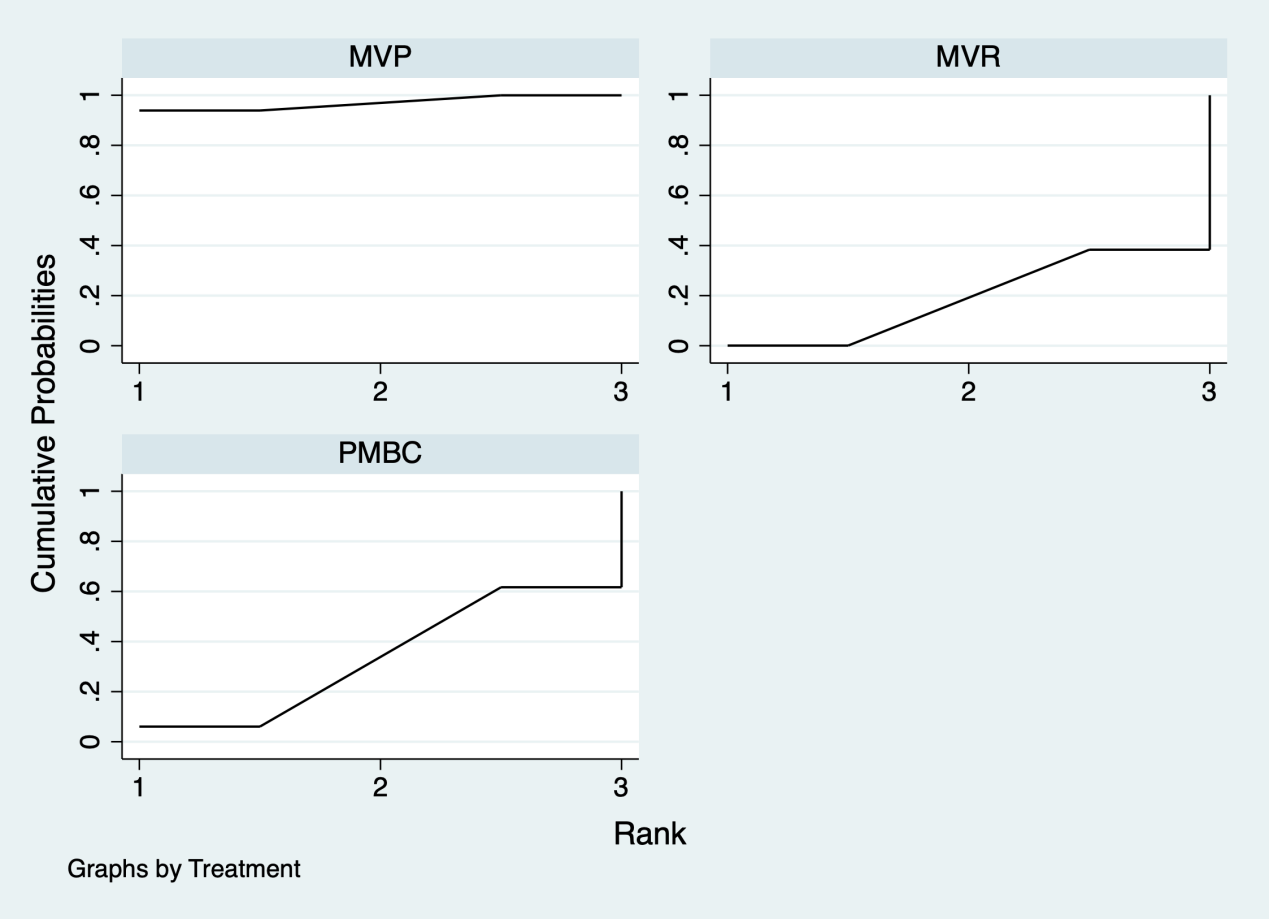


Figure S6. Plots of cumulative ranking probability on follow-up mortality. (SUCRA)


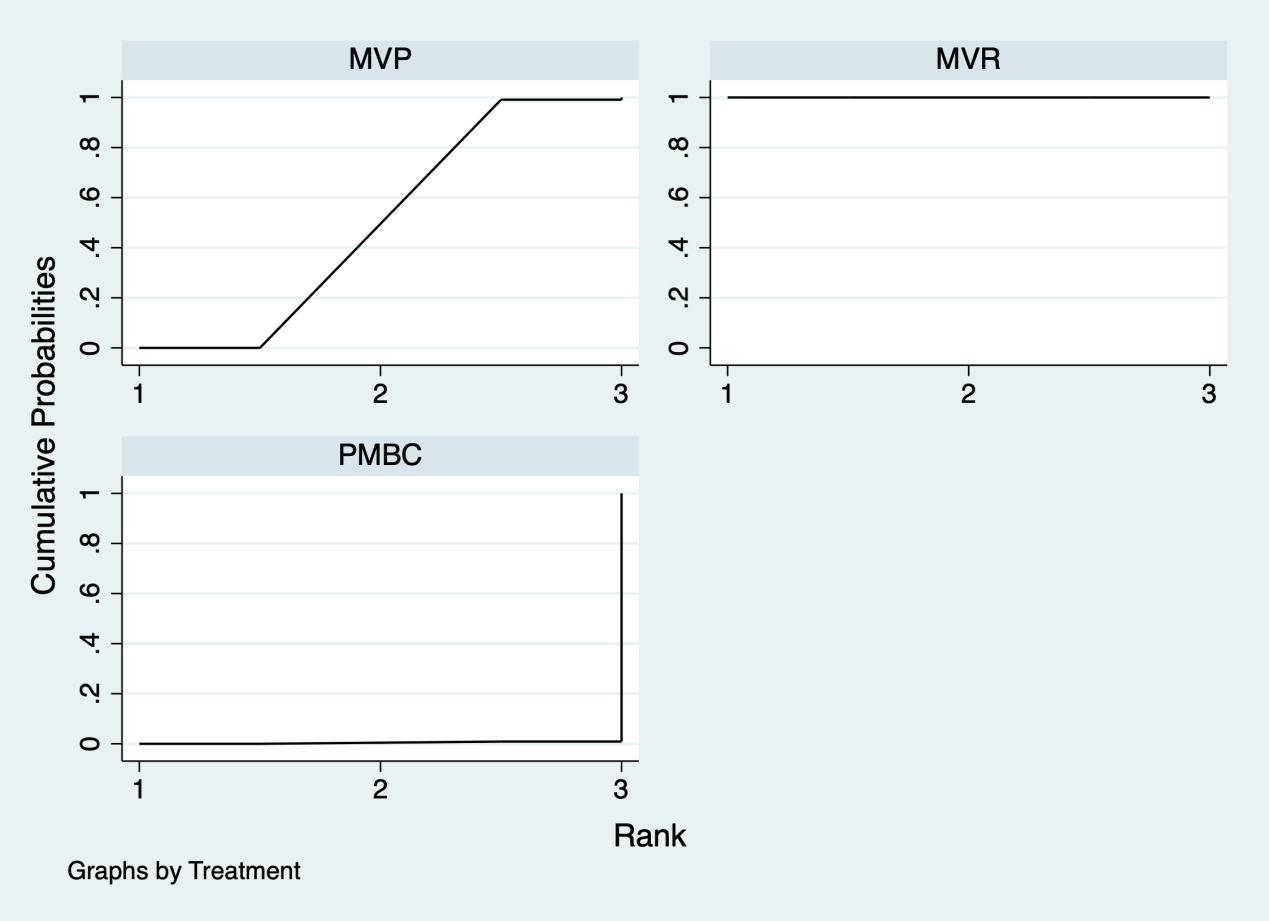


Figure S7. Plots of cumulative ranking probability on follow-up reoperation. (SUCRA)


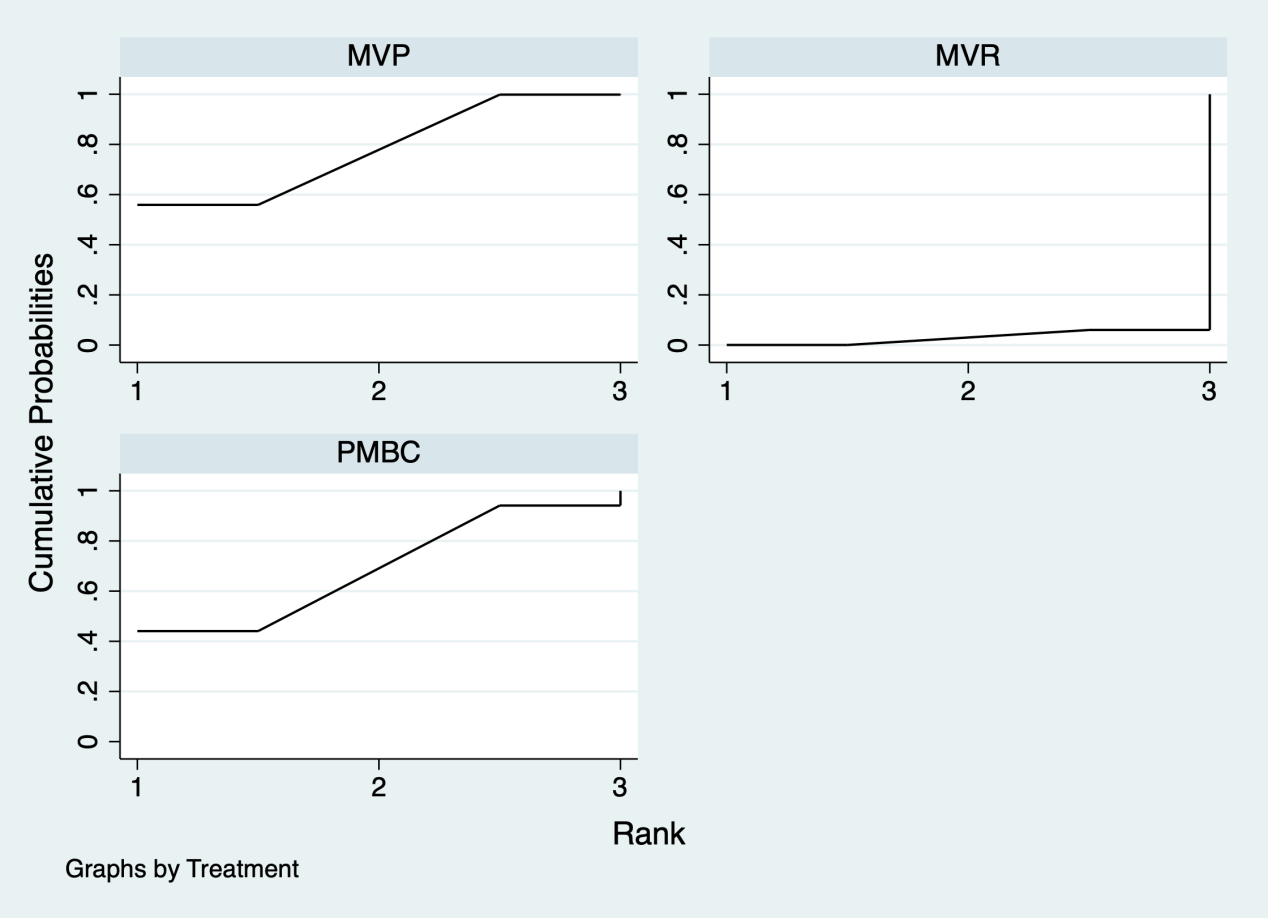


Figure S8. Plots of cumulative ranking probability on follow-up complications. (SUCRA)


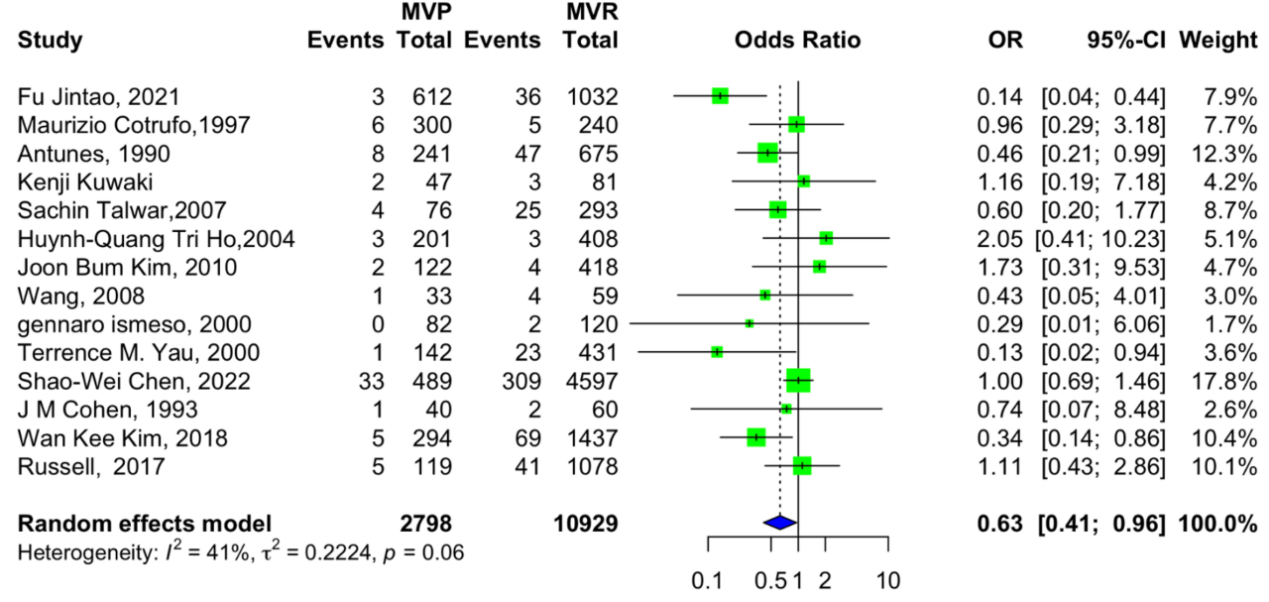


Figure S9. Early mortality between MVP vs MVR.


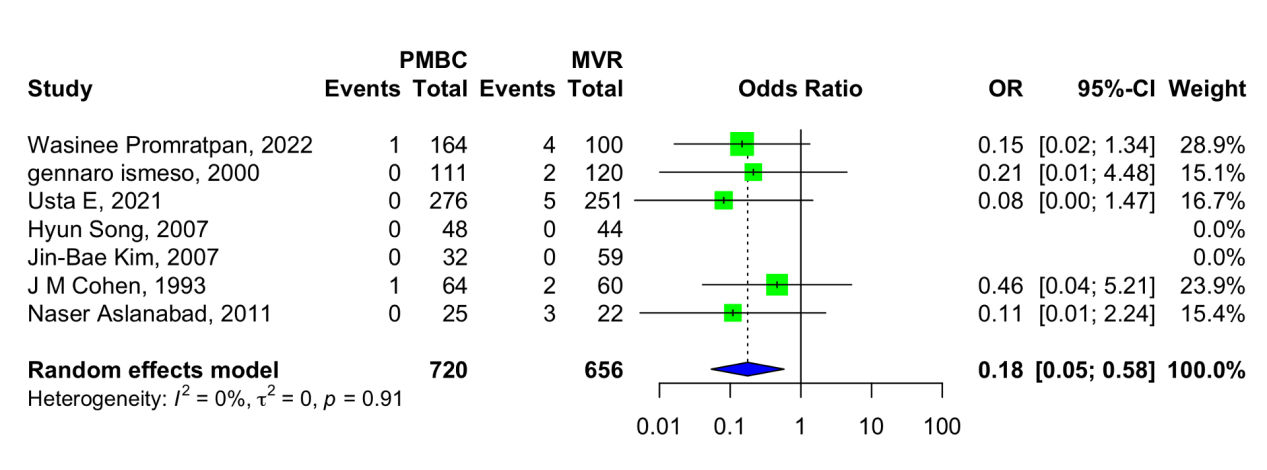


Figure S10. Early mortality between PMBC vs MVR.


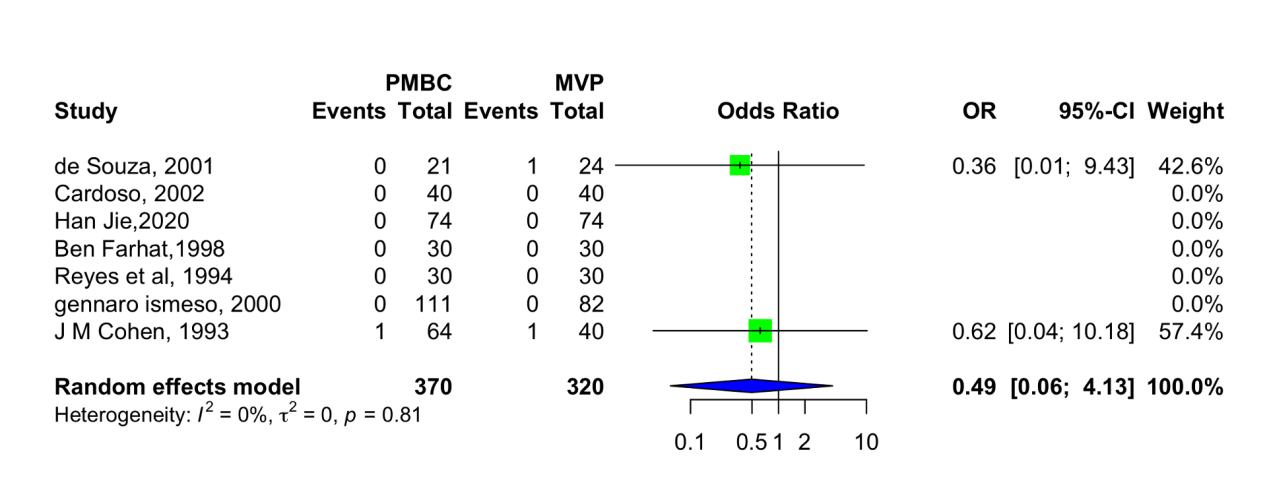


Figure S11. Early mortality between PMBC vs MVP.


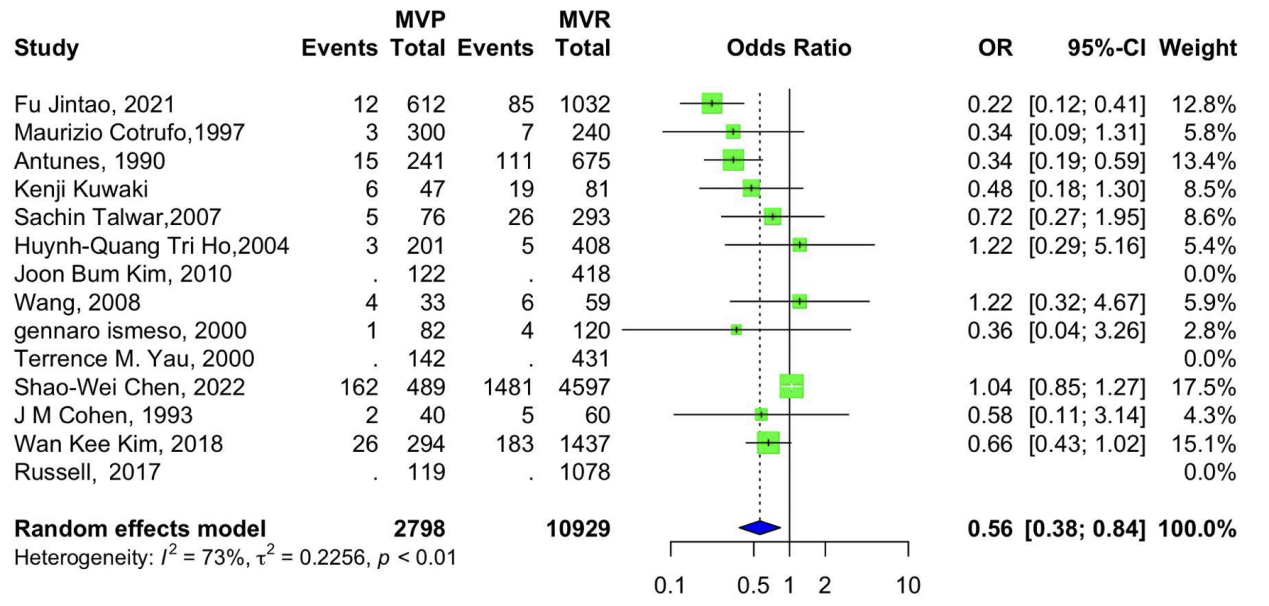


Figure S12. Follow-up mortality between MVP vs MVR.


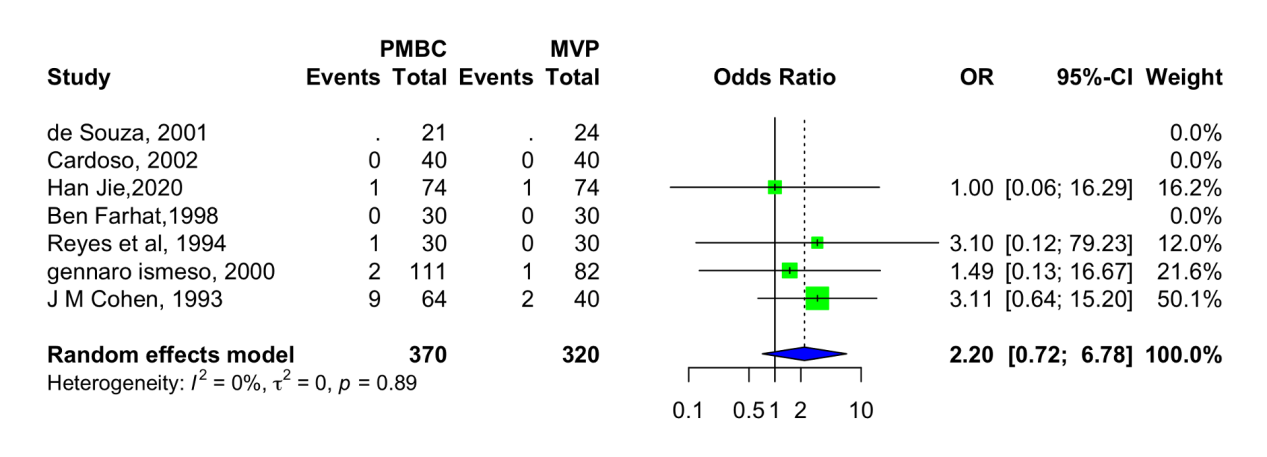


Figure S13. Follow-up mortality between PMBC vs MVP.


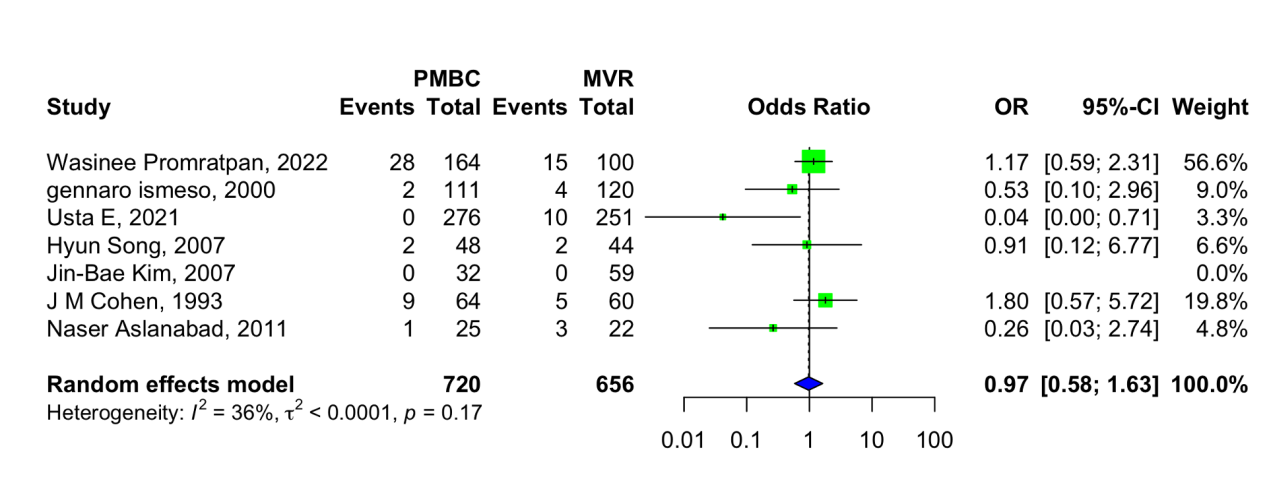


Figure S14. Follow-up mortality between PMBC vs MVR.


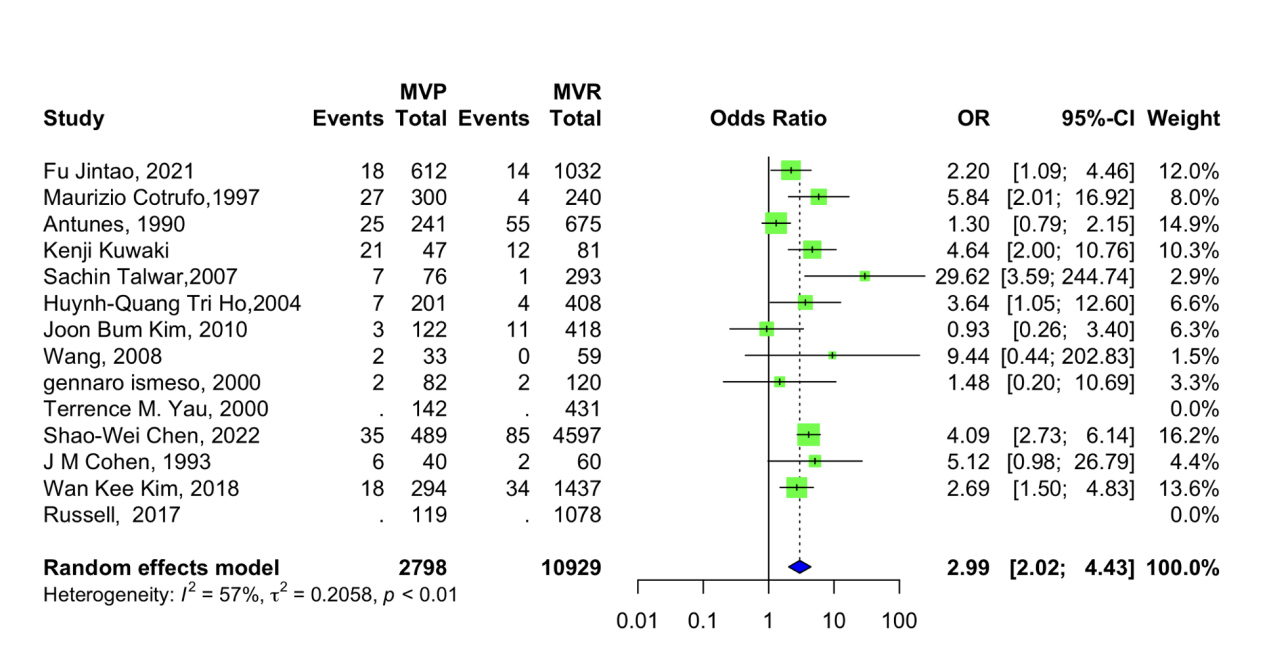


Figure S15. Follow-up reoperation between MVP vs MVR.


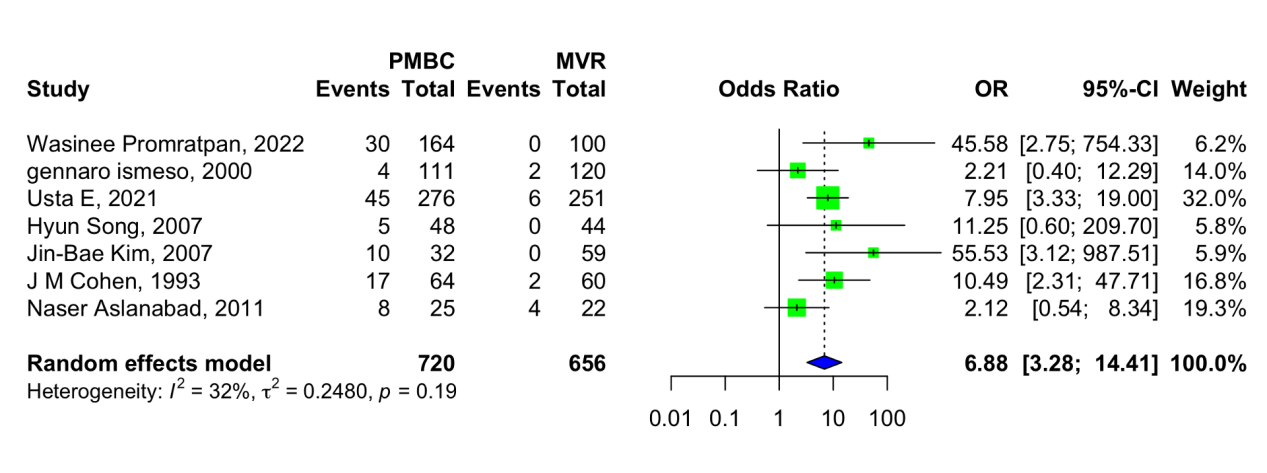


Figure S16. Follow-up reoperation between PMBC vs MVR.


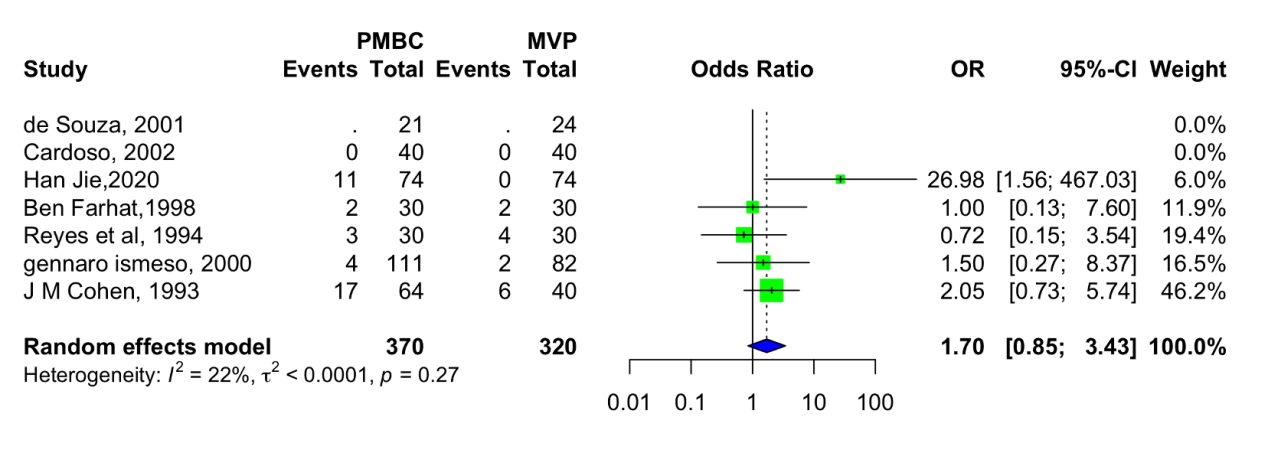


Figure S17. Follow-up reoperation between PMBC vs MVP.


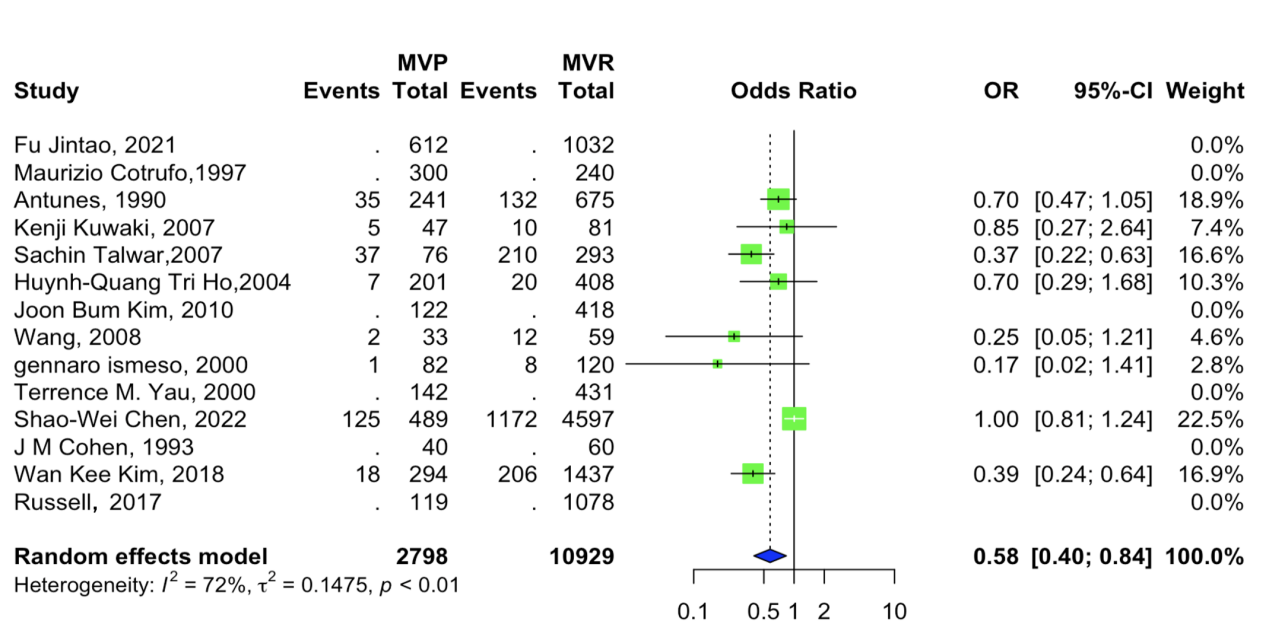


Figure S18. Follow-up complications between MVP vs MVR.


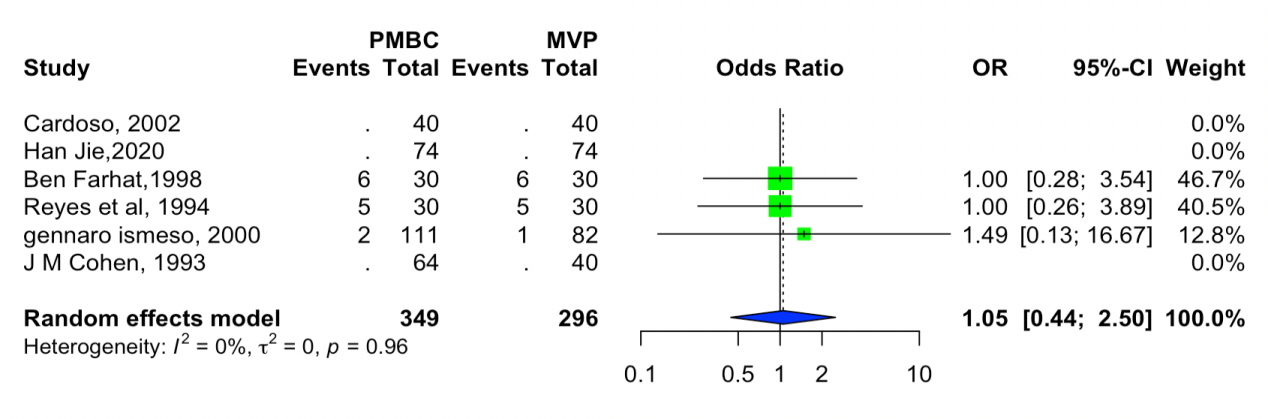


Figure S19. Follow-up complications between PMBC vs MVP.


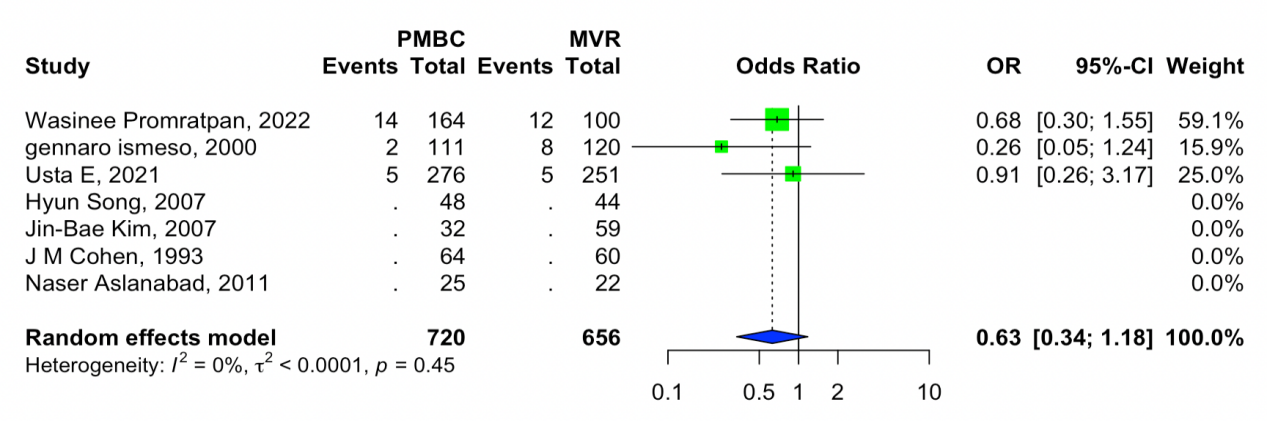


Figure S20. Follow-up complications between PMBC vs MVR.


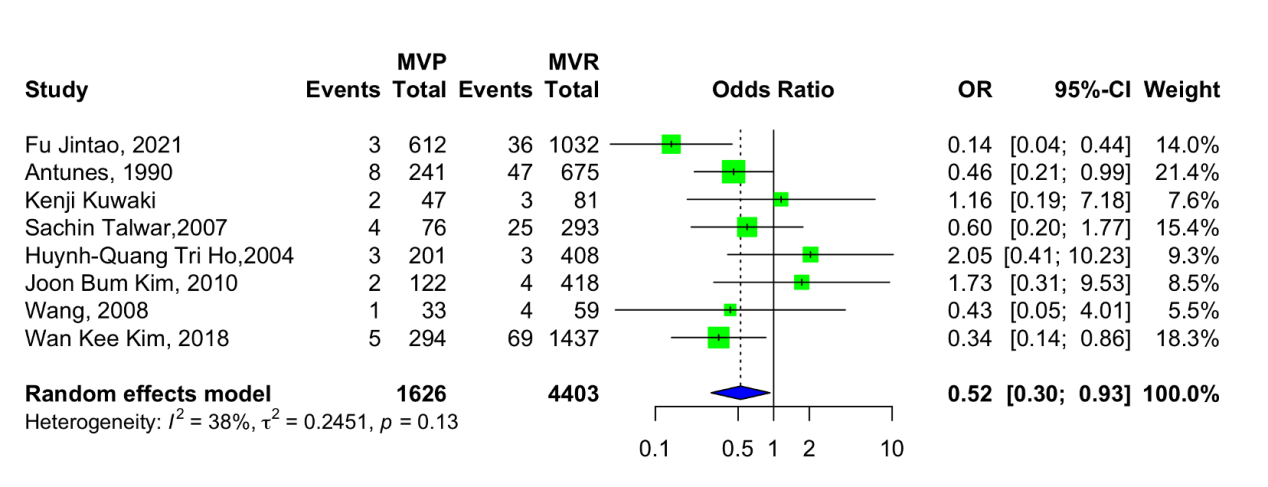


Figure S21. Early mortality between composite MVP vs MVR. (subgroup analysis)


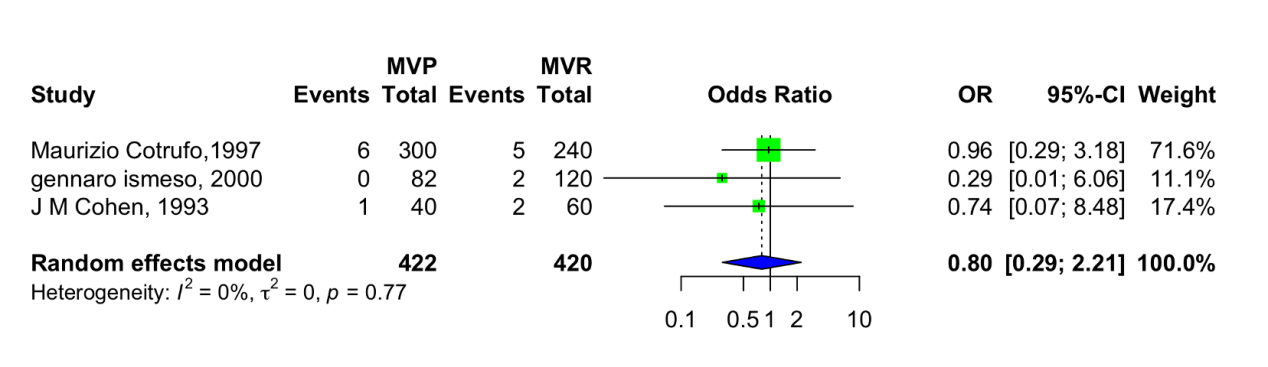


Figure S22. Early mortality between simple commissurotomy vs MVR. (subgroup analysis)


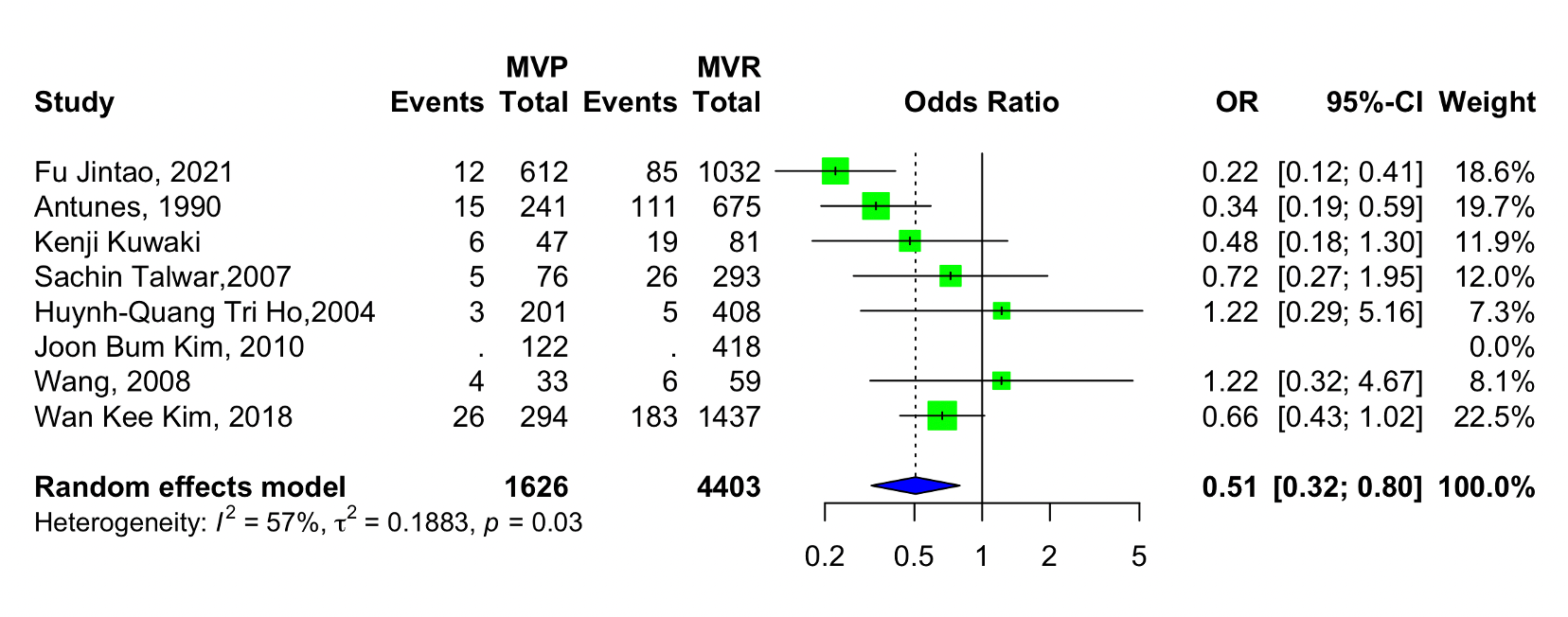


Figure S23. Follow-up mortality between composite MVP vs MVR.


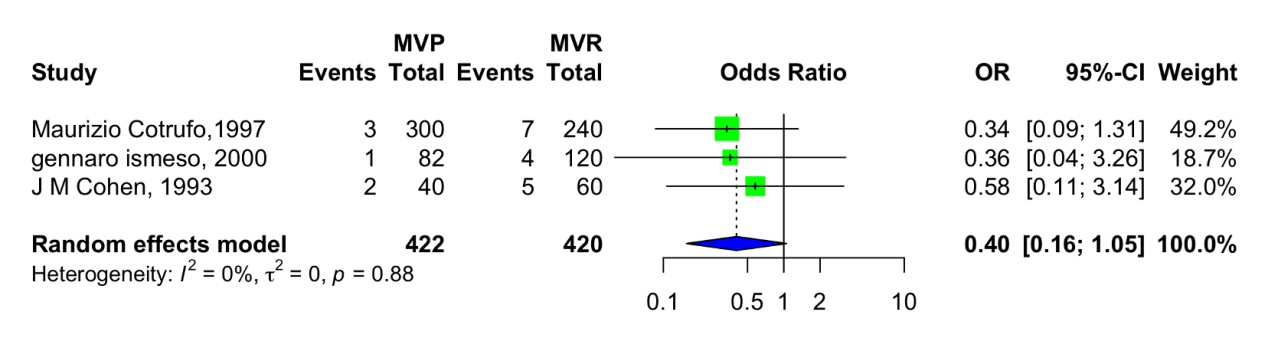


Figure S24. Follow-up mortality between simple commissurotomy vs MVR.


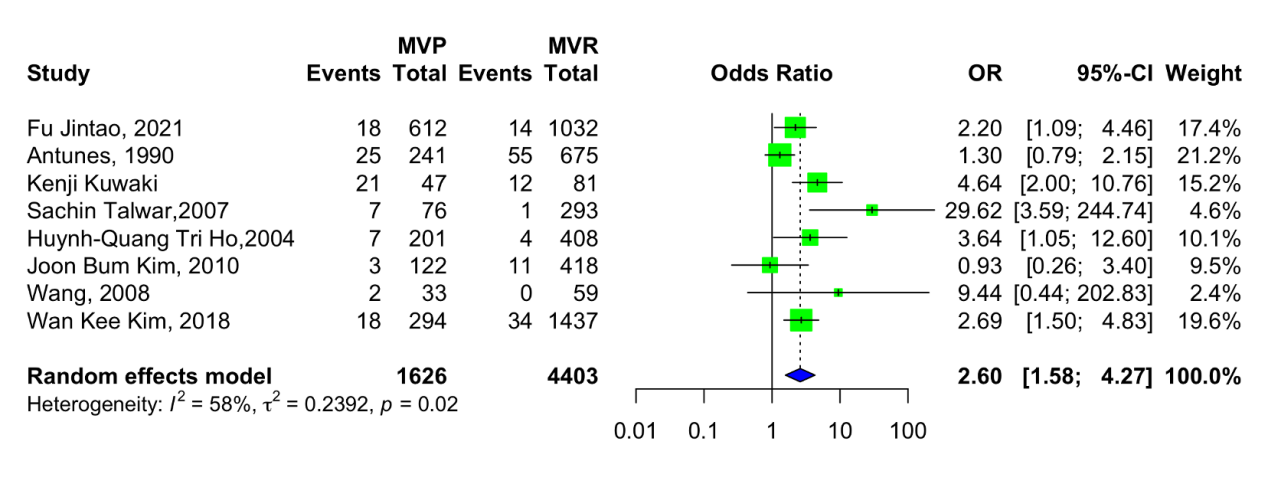


Figure S25. Follow-up reoperation between composite MVP vs MVR.


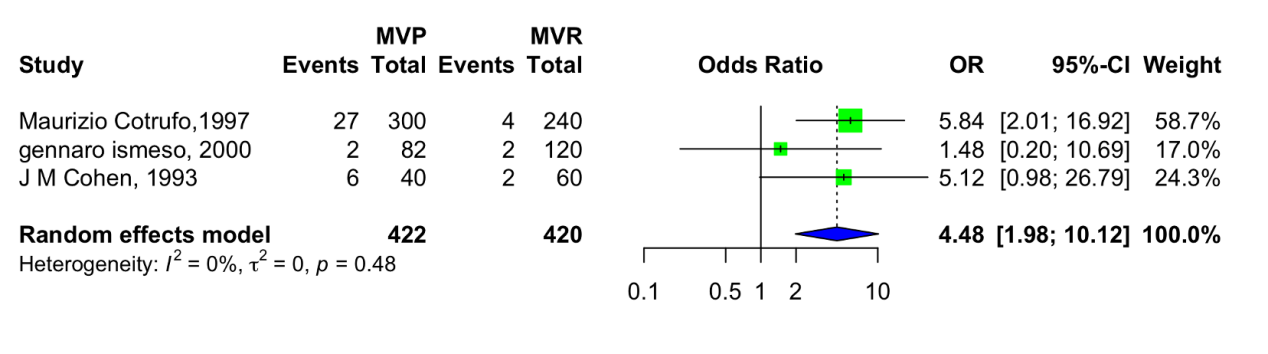


Figure S26. Follow-up reoperation between simple commissurotomy vs MVR.


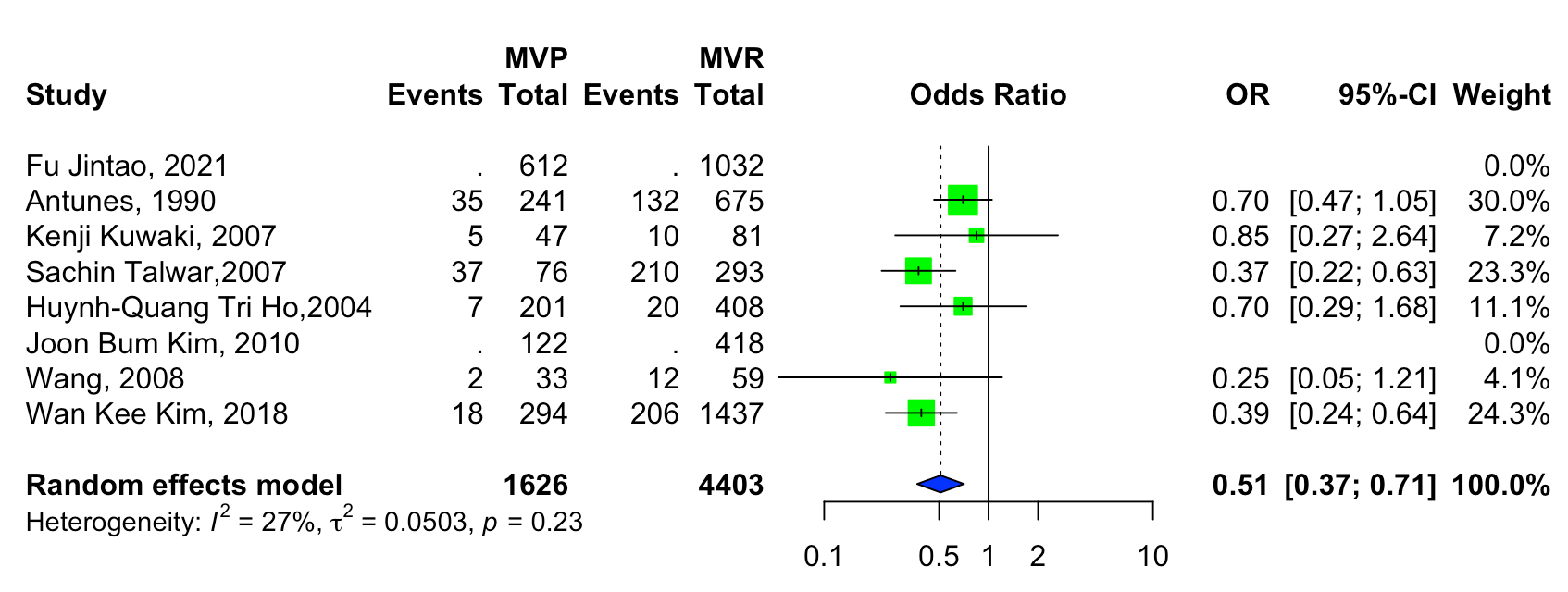


Figure S27. Follow-up complications between composite MVP vs MVR.


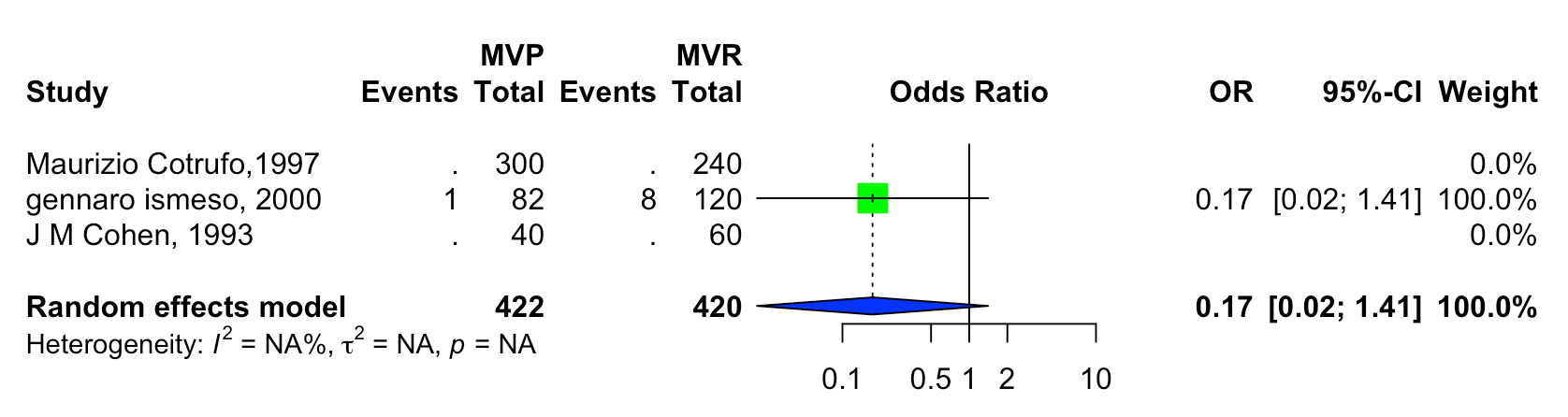


Figure S28. Follow-up complications between simple commissurotomy vs MVR.
